# Assessing resistome host range across water reclamation in three geographically distinct communities using Hi-C sequencing

**DOI:** 10.1128/aem.00701-26

**Published:** 2026-07-27

**Authors:** Sarah E. Philo, Michael A. Saldana, Harmita Golwala, Siyi Zhou, Jeseth Delgado Vela, Lauren B. Stadler, Adam L. Smith

**Affiliations:** 1 Sonny Astani Department of Civil and Environmental Engineering, University of Southern California 5116, Los Angeles, California, USA; 2 Department of Civil and Environmental Engineering, Rice University 3990, Houston, Texas, USA; 3 Department of Civil and Environmental Engineering, Duke University 3065, Durham, North Carolina, USA; Indiana University Bloomington, Bloomington, Indiana, USA

**Keywords:** antimicrobial resistance, proximity ligation, Hi-C, shotgun sequencing

## Abstract

Antimicrobial resistance (AMR) is a growing problem, with annual deaths set to pass 10 million by 2050 if current trends continue. Sequencing untreated wastewater has been proposed as a strategy to understand population-level resistance, and water reclamation facilities (WRFs) have been identified as a control point for environmental dissemination of resistant bacteria. Understanding AMR dynamics across WRFs can provide insight into how WRFs may alter the resistome and requires advanced molecular tools that elucidate host bacteria, especially for mobile resistance carried on plasmids. To that end, influent, activated sludge, and effluent were collected from three WRFs in North Carolina, Texas, and California during 3 weeks of spring 2024. Samples were analyzed for chromosomal and, crucially, plasmid-based resistance using Hi-C proximity ligation sequencing to identify the AMR host range. A total of 1,868 hits for 244 unique resistance genes were observed, with seven resistance genes identified in all samples: *bla_OXA_, merE, P, R,* and *T*, *msr(E*), and *tet(C*). Resistance genes within microbial hosts were more likely to be carried on a plasmid in influent, but more likely to be on a chromosome in activated sludge. Seventeen total microbial hosts for resistance genes were identified in effluent, with a *Moraxellaceae* sp. and *Thiobacillaceae* sp. carrying ARGs on plasmids. More than 50% of all identified host relationships were confined to just four bacterial families: *Aeromonadaceae, Burkholderiaceae, Moraxellaceae,* and *Neisseriaceae*. Hi-C contact mapping is a critical tool to more fully describe the AMR host range in complex matrices, particularly for plasmid-based resistance genes.

**IMPORTANCE:**

Water reclamation facilities receive a complex mixture of antibiotics and rely on active microbial communities for treatment, thereby acting as critical systems to prevent environmental spread of resistance. However, antimicrobial resistance (AMR) dynamics are difficult to discern in complex wastewater environments due to antibiotic resistance genes (ARGs) being frequently carried on mobile pieces of DNA that are difficult to link to specific bacteria using conventional shotgun sequencing. In the current study, Hi-C sequencing was carried out on influent, activated sludge, and effluent collected from water reclamation facilities in California, Texas, and North Carolina to assess the resistome host range across treatment. The current study elucidated differences in the genomic context of ARGs across treatments, showing that ARGs are more likely to be plasmid-based in influent bacterial communities but located on chromosomes in activated sludge. Hi-C sequencing is uniquely able to provide untargeted linkage to plasmids with their microbial hosts in complex communities

## INTRODUCTION

Antimicrobial resistance (AMR) is one of the greatest threats to public health, with AMR-associated deaths predicted to increase to 10 million deaths annually by 2050 from 4.9 million deaths in 2021 if growing resistance is not addressed (1). Despite increased attention to AMR, new resistance patterns are emerging worldwide. Most notably, plasmid-mediated colistin resistance (*mcr-1*) emerged in the early 2010s in China (2), and carbapenem-resistant Enterobacteriaceae (*bla*_NDM-1_) emerged in the early 2000s in India and Pakistan (3). Influent wastewater collected from water reclamation facilities (WRFs) has been proposed as a suitable sample to monitor population-level AMR because it is representative of the wastewater catchment population (4–6). WRFs are also known as wastewater treatment plants. Given the recent movement to recognize the recoverable resources in wastewater, we will be using the term WRFs. Assessing AMR across facilities and wastewater catchment areas is challenging due to the lack of standardized treatment processes and variability in influent wastewater composition, with differing contributions from domestic, agricultural, and industrial sources. To help facilitate geographic comparisons by looking at shared antibiotic resistance genes (ARGs), a recent global study of influent wastewater identified 13 ARGs in every sample collected from 243 cities in 101 countries (i.e., the core resistome [7]), in addition to 127 ARGs in at least half of the samples (8). This suggests that wastewater can be used to identify resistome similarities across geography. AMR surveillance across WRFs is additionally critical as these systems are considered antibiotic and antimicrobial resistance “hotspots” due to the combination of microbial communities, antibiotics, and disinfectants (9–11). ARGs are routinely detected in WRF effluents (12), making WRFs both sources of and critical control points to mitigate dissemination of resistance to the environment (13). Furthermore, surface waters downstream of WRFs have been found to have higher ARG abundance relative to upstream surface waters (14–16), indicating the facilities themselves are sources of resistance in the environment.

There are numerous open questions about how different treatment processes in WRFs select for or remove AMR. A review by C. X. Hiller et al. (17) summarized the efficacy of different wastewater treatment processes, noting that antibiotic-resistant bacteria (ARB) and ARGs often have different removal rates from the same treatment process. Critically, existing research does not identify if and how the bacteria carrying ARGs (i.e., the ARG host range) changes during treatment. This is particularly relevant for non-culturable ARBs for whom it is nearly impossible to determine resistance phenotypes. WRFs are designed to meet specific pathogen reduction values without considering ARB in effluents. Identifying drivers of the AMR host range throughout treatment and in effluents could decrease environmental risks of releasing ARBs.

Horizontal gene transfer (HGT) of resistance genes via mobile genetic elements (MGEs) and plasmids is particularly concerning in WRFs due to the complex microbial communities in the presence of various selective pressures (11). Understanding microbial hosts of resistance genes on plasmids and their evolution is therefore crucial. Yet, shotgun metagenomic sequencing for wastewater-based AMR surveillance can only identify chromosomal microbial hosts of ARGs (4, 13, 18). The inability to link plasmid ARGs with host microbes remains a substantial weakness of shotgun sequencing to study AMR dynamics. Single-cell sequencing techniques like epicPCR have been developed to overcome these limitations (19). However, this method is a targeted approach limited to pre-chosen genes, preventing holistic analyses. Novel chromosome conformation capture (3C) techniques, such as Hi-C, link ARGs on plasmids to their host microbes by crosslinking physically close DNA in a non-targeted manner prior to cell lysis (20). Hi-C sequencing utilizes both the crosslinked sequenced DNA and a traditional shotgun sequencing assembly to obtain results. Resistance gene annotation and taxonomic assignment are carried out on the traditional shotgun sequences. The crosslinked DNA is then used to bin shotgun contigs into metagenome-assembled genomes (MAGs) and plasmid MAGs (pMAGs) and to assign microbial hosts to identified resistance genes and pMAGs. These 3C techniques remain the only non-targeted methods to identify microbial-plasmid-ARG relationships, removing a substantial limitation of traditional shotgun sequencing for AMR surveillance. Hi-C is an established method and has been employed to study AMR in untreated wastewater (20, 21) and across wastewater treatment to reconstruct plasmid and ARG networks in these matrices (22). However, previous work assessing AMR utilizing Hi-C is limited in geographic and WRF diversity, which is required to more fully understand plasmid AMR dynamics in complex environmental matrices.

Numerous questions remain about AMR dynamics through different treatment trains, across geographic distance, and following disinfection. In this study, we used Hi-C proximity-ligation sequencing to test the following hypotheses by collecting influent, activated sludge (AS), and effluent from WRFs in California (CA), Texas (TX), and North Carolina (NC) for 3 weeks in Spring 2024. The CA WRF treats over 400 million gallons a day (MGD) of wastewater with both residential and industrial input streams. The TX WRF treats over 80 MGD of primarily residential wastewater. The NC WRF treats approximately 10 MGD of wastewater composed primarily of residential waste with some industrial. Samples were collected from these locations to highlight the need for geographic and wastewater catchment diversity in AMR studies. Additionally, each facility has different treatment processes. NC uses a modified five-stage Bardenpho process with UV for final disinfection, whereas both TX and CA use high-purity O_2_-activated sludge and chlorine to disinfect final effluent, but TX does not have primary clarification and additionally filters their final effluent. We hypothesized that (i) the relative proportion of all ARGs with hosts will decrease across treatment, (ii) effluent bacterial hosts will be dependent on different final treatment processes in the three sampling locations, and (iii) a few bacterial families will be responsible for the bulk of the host relationships. We assessed AMR, ARG host range, and changes in the resistome during wastewater treatment in the three WRFs sampled. Additionally, we assayed effluent to assess the potential to release clinically relevant ARB from WRFs.

## RESULTS

### Bacterial community structure

**Fig 1:**
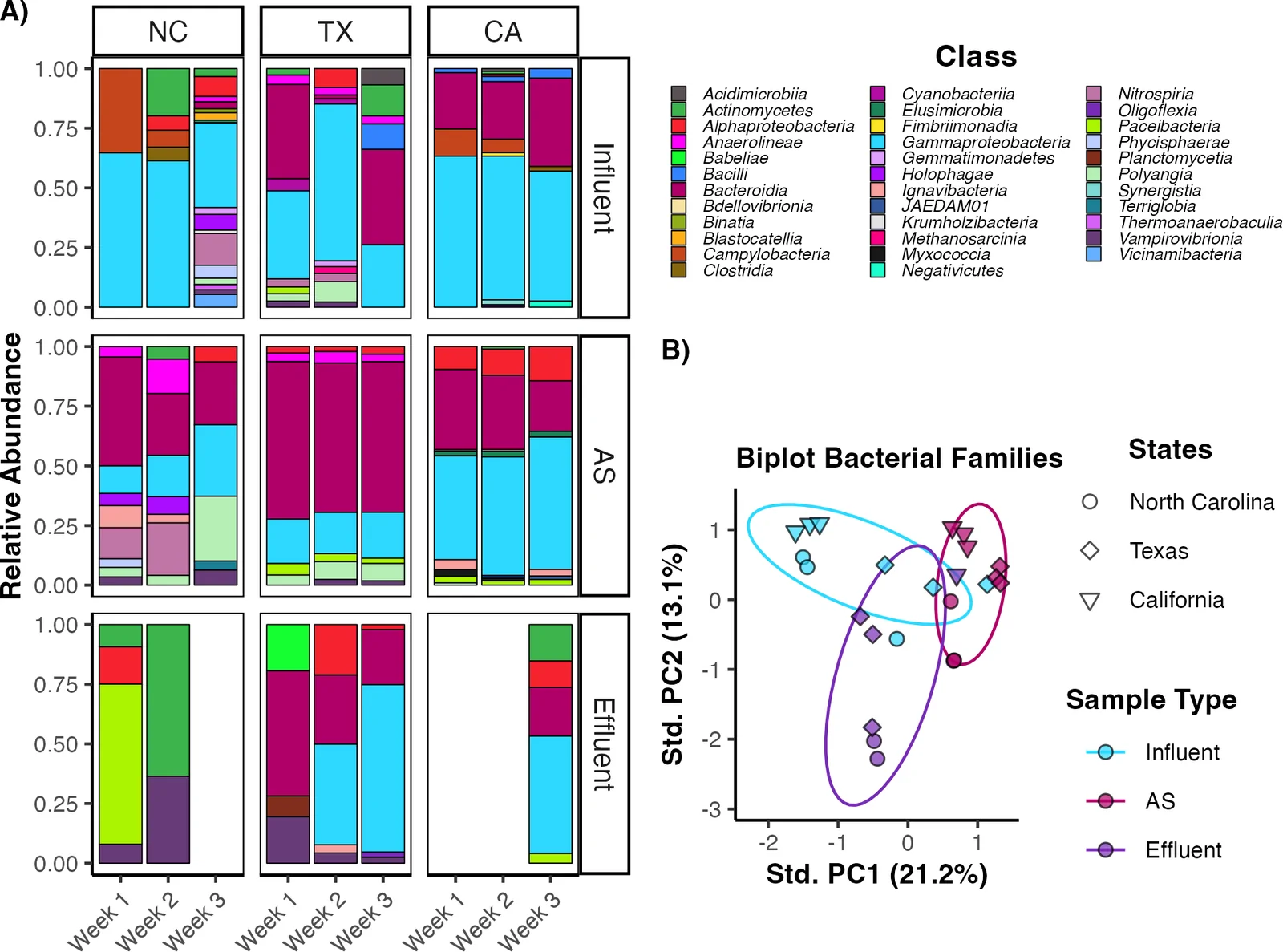
(**A**) Relative abundance of bacterial classes for each sampling location, sample type, and sampling event of the MAGs. MAGs were filtered for >70% completion and <20% contamination. Relative abundance was calculated by adjusting transcripts per million (TPM) to the microbial fraction of the shotgun sequencing data. Effluent samples from NC and CA are not included because they could not undergo the full sequencing pipeline due to low mapping of Hi-C data to shotgun assemblies. (**B**) Biplot of PCA with centering and scaling for bacterial family relative abundance following a Hellinger transformation of MAGs. Ellipses represent 68% coverage of the data for each sample type collected across geographic locations. PERMANOVA identified significant clustering at both the state level (*P* < 0.001) and sample type (*P* < 0.001). NC, North Carolina; TX, Texas; CA, California; AS, activated sludge.

Relative abundance of bacterial MAGs (>70% completion, <20% contamination) was assessed at the class and family taxonomic levels (Fig. 1). MAGs were used to understand community structure because there is higher confidence in the sequencing quality and taxonomic assignment relative to the assemblies and bins generally had fairly high coverage of the assemblies (Table S1). Additionally, MAGs were used to identify microbial hosts. Results are only reported for six out of nine effluent samples collected, as the full analysis pipeline could not be completed due to poor mapping of the Hi-C data to the assemblies. The influent microbes in CA were highly stable week to week, while communities in NC and TX influent were less stable (Fig. 1). The influent microbial communities in all three were dominated by *Gammaproteobacteria* (mean relative abundance 52%, 95% CI: 41%–64%). However, microbial communities in AS from all three locations were highly stable week to week, exhibited strong similarity across geography, and were dominated by *Bacteroidia* spp. (mean relative abundance 42%, 95% CI: 28%–56%; Fig. 1). Effluent communities exhibited little similarity to each other over time or to the AS in all three sampling locations (Fig. 1). Significant clustering in microbial composition was identified with PERMANOVA at both the state level (*P* < 0.001) and sample type (*P* < 0.001).

### Resistome assessment

AMR was assessed at different levels (Fig. S1). Here, total resistance genes were defined as the total count of resistance genes for drugs, heavy metals, and environmental stressors identified in AMRFinderPlus. There were 1,868 total resistance genes detected in the shotgun sequencing assemblies (Table 1; Fig. S1 and supplemental material, section 3.2). Total ARGs were defined as the subset of total resistance genes encoding efflux pumps and conferring resistance to antibiotic drugs (*n* = 955, Table 1). Unique total resistance genes and ARGs consisted of each individual gene detected and counted once. There were 244 unique total resistance genes and 173 unique ARGs (Fig. S1). The number of total resistance genes detected was highest in the influent (*n* = 1,099) compared to AS (*n* = 414). ARGs similarly saw a reduction in detection from influent (*n* = 601) to AS (*n* = 192). Across all samples and gene types, the bulk of the reduction in individual genes occurred between the influent and AS (Table 1). Total resistance genes increased between AS and effluent in each week sampled for which there was an effluent sample (Table 1).

**TABLE 1:** Total number of resistance genes detected in each sampling location at each treatment level^*a*^

		North Carolina (*n* = 8)		Texas (*n* = 9)		California (*n* = 7)			Total (*n* = 24)	
		ARGs	Total resist.	ARGs	Total resist.	ARGs	Total resist.		ARGs	Total resist.
Week 1	Influent	94	169	34	69	62	115	**Influent**	**601**	**1,099**
	AS	13	24	20	50	18	43			
	Effluent	16	38	23	54	0	0			
Week 2	Influent	94	158	43	81	97	170	**AS**	**192**	**414**
	AS	12	22	26	60	23	43			
	Effluent	24	49	47	78	0	0			
Week 3	Influent	35	82	55	92	87	163	**Effluent**	**162**	**355**
	AS	31	68	26	58	23	46			
	Effluent	0	0	23	59	29	77			
**Total**		**319**	**610**	**297**	**601**	**339**	**657**		**955**	**1,868**

^*a*^ ARGs are representative only of resistance genes that encode drug resistance or efflux pumps. Total resistance (resist.) includes both ARGs and heavy metal/environmental stressor resistance genes. The total number of samples analyzed at each location is the *N* value. AS, activated sludge. Bold formatting indicates the total values across rows and columns.

The entire resistome, including resistance genes for drugs, efflux pumps, heavy metals, and environmental stressors, was assessed to understand how host carriage changes over treatment. When considering potential host bacteria, Hi-C sequencing identified plasmid-based hosts, chromosomal-based hosts, or no host. Hosts were assigned based on identification in a microbial genomic bin. In the event that no host was assigned, it was due to either (i) no informative Hi-C crosslinks between microbial bins and contigs containing resistance genes or (ii) unbinned contigs leading to a lack of host identification for resistance genes (mean bin coverage = 42.4%, Table S1), which is a shared challenge with traditional shotgun sequencing analysis. 12.3% of all detected resistance genes were identified in a microbial host, with 7.4% carried in microbial plasmids and 4.9% carried in microbial chromosomes. While the majority of detected resistance genes did not have a microbial host identified across all sample types and locations, resistance genes were more likely to be in a microbial plasmid in influent and in a chromosome in AS (Table 2). When considering only ARGs, they were more likely to be in a plasmid in influent wastewater, but no clear trend was observed in AS (Table S2). Very few microbial hosts of resistance genes were identified in effluent samples (Table 2; Table S2), despite increased detection of total resistance genes from AS to effluent in weeks where both samples underwent the full sequencing pipeline (Table 1).

**TABLE 2:** Total resistance genes detected in either a bacterial host plasmid or chromosome by Hi-C contact mapping across all samples that underwent the complete deconvolution platform^*a*^

	North Carolina			Texas			California		
	*n*_tot_	Plasmid	Chr.	*n*_tot_	Plasmid	Chr.	*n*_tot_	Plasmid	Chr.
Influent	409	43	12	242	28	3	448	42	15
		10.5%	2.90%		11.50%	1.20%		9.40%	3.30%
AS	114	6	13	168	5	7	132	11	28
		5.30%	11.40%		3.00%	4.20%		8.30%	21.20%
Effluent	87	0	0	191	0	4	77	4	9
		0%	0%		0%	2.10%		5.20%	11.70%

^*a*^ *n*_tot_ represents the total number of genes detected in that sample type from that geographic area. Percentages represent the fraction of genes detected in either a microbial plasmid or chromosome (Chr.) from that sample type and geographic location. AS, activated sludge.

**Fig 2:**
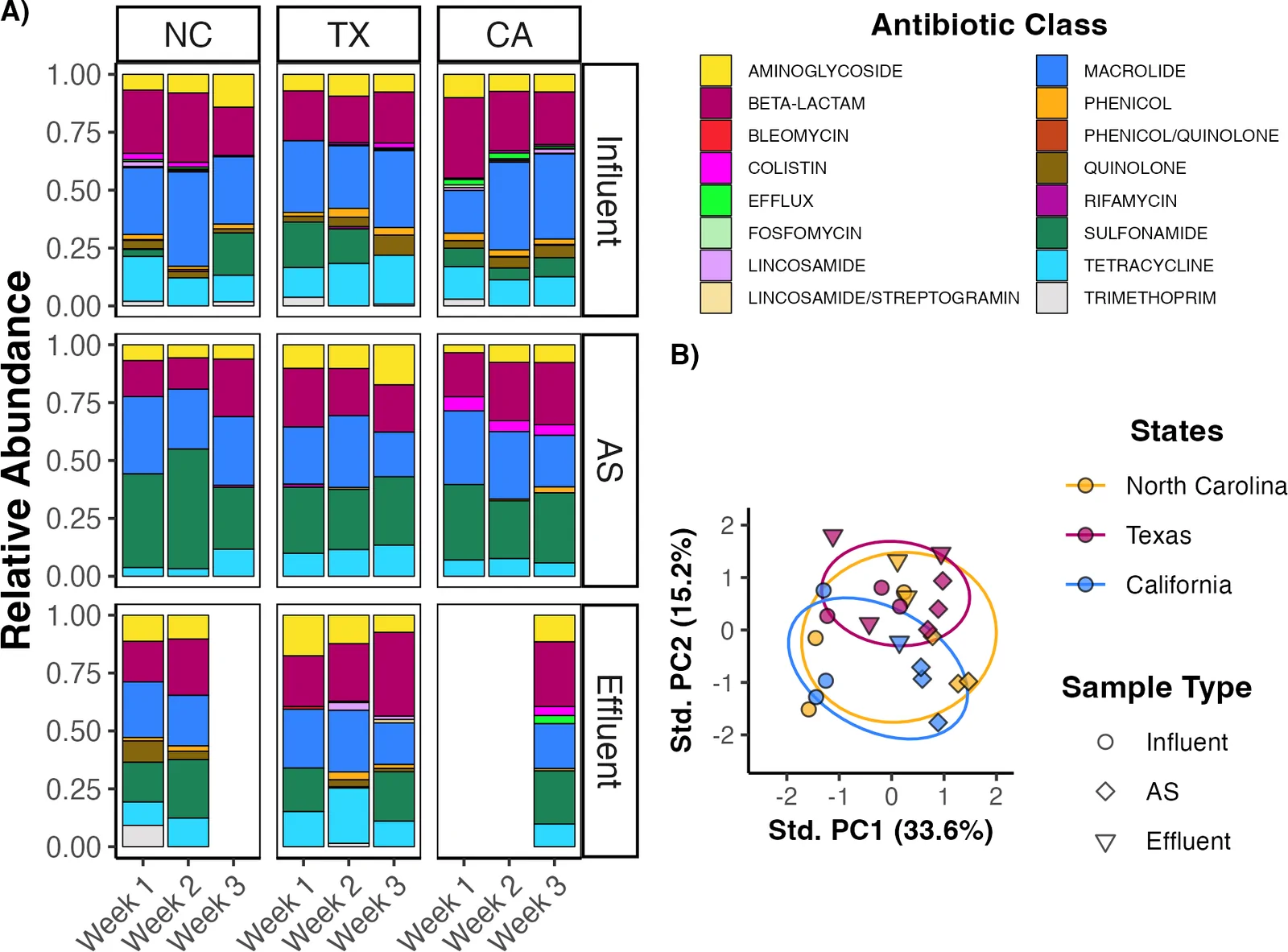
(**A**) Relative abundance of antibiotic classes over treatment and time. Relative abundance was calculated by adjusting TPM for the ARG fraction of the shotgun sequencing data. Contigs were filtered for ARG sequences that confer resistance to pharmaceuticals. Effluent samples in NC and CA are not included because they could not undergo the full sequencing pipeline due to low mapping of Hi-C data to assemblies. (**B**) Principal component analysis with centering and scaling of the resistome relative abundance at the antibiotic class level following Hellinger transformation. Ellipses represent 68% coverage of the data for samples collected from each state. PERMANOVA identified significant clustering among sample types (*P* < 0.001), but not at the state level (*P* = 0.111). NC, North Carolina; TX, Texas; CA, California; AS, activated sludge.

Among the ARGs, the most commonly detected antibiotic class across all samples collected was beta-lactams (*n* = 320), followed by macrolides (*n =* 180), tetracycline (*n* = 138), and aminoglycoside (*n* = 112) (Table S3). When considering relative abundance, macrolide resistance and beta-lactam resistance were the most abundant on average across all samples, with not statistically different relative abundances of 27.7% (95% CI = 25.1%–30.4%) and 23.7% (95% CI = 21.5%–26.0%), respectively (Fig. 2A; Table S3). To assess resistome similarity across sampling locations, PCA followed by PERMANOVA was carried out on the relative abundance of antibiotic classes (Fig. 2B; Fig. S2). There was significant clustering by sample identified by PERMANOVA type (*P* < 0.001; Fig. S2). The resistome of the AS exhibited the smallest variability, as evidenced by the smallest ellipse of the three sample types (Fig. S2). The influent resistome had the highest variability, as evidenced by the largest ellipse. PCA did not identify any unique resistome clusters at the state level with PERMANOVA (*P* = 0.11) as all sample ellipses overlapped with each other (Fig. 2B). Resistance to specific antibiotic classes stayed relatively stable over time, between sampling locations, and across treatment processes (Fig. 2). Additionally, even though different secondary treatment processes are employed at NC (five-stage Bardenpho) and TX and CA (high-purity O_2_ activated sludge), the resistance gene abundance in the AS from all three WRFs was highly similar (Fig. S2).

### Core resistome

**Fig 3:**
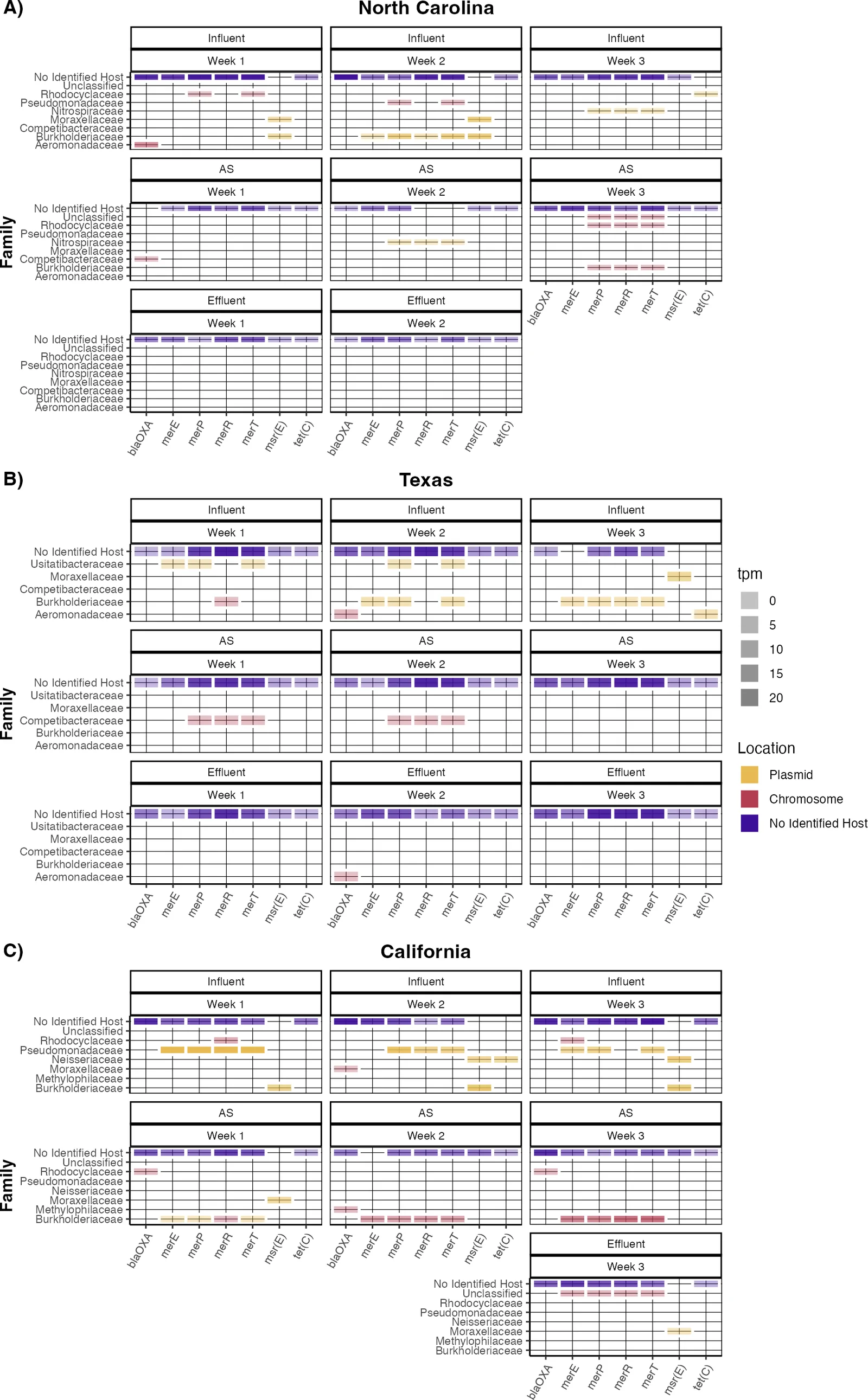
Hosts of the core resistome for each sample collected in (**A**) North Carolina, (**B**) Texas, and (**C**) California. The core resistome is defined as the individual resistance genes detected in every sample collected in this study. Color represents the location in the bacterial genome (either plasmid or chromosome), and the shading represents the unadjusted TPM value. “No Identified Host” were resistance genes annotated on contigs with no informative Hi-C crosslinks or insufficient sequencing depth or quality to identify a bacterial host bin. AS, activated sludge.

To observe how resistance genes move through microbial hosts in WRFs, the core resistome (i.e., resistance genes detected in all samples) was identified. Seven resistance genes were identified in the core resistome: *bla*_OXA_*, merE, merP, merR, merT, msr(E),* and *tet(C)* (Table S4; Fig. 3). They were detected a total of 505 times with 102 host relationships. Four genes, *merE, merP, merR,* and *merT,* encode mercury resistance in the *mer* operon (23, 24), working together via disparate functions to detoxify mercury. The remaining three genes in the core resistome, *bla*_OXA_*, msr(E),* and *tet(C*) encode resistance to drugs. *bla*_OXA_ encompasses a diverse group of genes encoding class D β-lactamases and confers resistance against later-generation cephalosporins and carbapenem (25). Additional *bla_OXA_* alleles detected were *bla_OXA-_*_1, 2, 4, 5, 9, 10, 119, 347, 732, and 912_, although no hosts were identified (supplemental material, section 3.1). Hi-C crosslinking identified *bla_OXA_* as being carried on bacterial chromosomes in *Aeromonas caviae* in NC and TX influent and TX effluent (Table S4 and supplemental material, section 3.1; Fig. 3). The *msr(E*) gene encodes macrolide resistance by interfering with the drug’s binding site on bacterial ribosomes (26, 27). Fifteen bacterial hosts were identified for *msr(E)* (Table S4). This gene was found on plasmids in *Burkholderiaceae* spp.*, Moraxellaceae* spp.*,* and *Neisseriaceae* spp. (Fig. 3). In NC influent, taxonomic assignment of bins identified an *msr(E*) host down to the species level: *Acinetobacter johnsonii* in the *Moraxellaceae* family (Fig. 3; supplemental material, section 3.1). Finally, *tet(C*) encodes an efflux pump conferring resistance to tetracycline (28–31) and was detected 24 times in the current study, with three plasmid-based bacterial hosts identified (Table S4).

### Location of resistance genes over treatment

To understand how the resistome is affected by treatment processes, the host range of resistance genes encoding drug resistance and efflux pumps (ARGs) detected in all influent samples of a single WRF (i.e., core influent ARGs) was assessed over treatment (Fig. S3 to S5; Table 3). There were 20 core influent ARGs detected in NC (Table 3). Most identified host-ARG relationships in the influent were plasmid-based (Fig. S3; Table 3). Among NC core influent ARGs, there were only two genes identified in the chromosome in the influent: *mph(F*) in *Rhodocyclaceae* and *bla*_OXA_ in *Aeromonadaceae* chromosomes (Fig. S3). In NC influent, *Burkholderiaceae* spp. contained three different macrolide resistance genes on plasmids: *mph(E)* (32), *msr(E)* (26, 27), and *mph(G)* (33) (Fig. S3). For NC core influent ARGs in AS, microbial hosts were only detected for *bla_OXA_* and *mph(A*), and these genes were carried in microbial chromosomes (Table 3; Fig. S3). No microbial hosts were identified in effluent (Table 3; Fig. S3).

**TABLE 3:** Location in bacterial hosts (plasmid or chromosome) for the core influent ARGs they move through the treatment process^*a*^

	North Carolina (20 shared ARGs)			Texas (14 shared ARGs)			California (36 shared ARGs)		
	No host ID'ed	Plasmid	Chr.	No host ID'ed	Plasmid	Chr.	No host ID'ed	Plasmid	Chr.
Influent	64	17	3	38	8	1	113	14	7
AS	20	0	3	26	2	0	28	3	8
Effluent	19	0	0	34	0	1	13	2	0

^*a*^ Core influent ARGs are resistance genes encoding efflux pumps or drug resistance that were detected in all influent samples within each water reclamation facility. Some ARGs were detected multiple times. Genes with no host identified (ID’ed) were annotated in the assembly but had no identified associations with bacterial bins or plasmids after processing Hi-C data. Chr., chromosome; AS, activated sludge.

TX had the fewest core influent ARGs detected (*n* = 14, Table 3), and most either did not have a host or were on a plasmid (Fig. S4). The exception to this was *bla*_OXA_, which was detected in the chromosome of an *Aeromonadaceae* spp. in the influent (Fig. S4). Of the TX core influent ARGs, most did not have a microbial host identified in TX AS (Table 3). However, two microbial hosts were identified in TX AS, *mph(A*) on a plasmid in a *Polyangiales* spp. and *tet(X2*) on a plasmid in an unclassified *Parvibacillus* (Fig. S4 and supplemental material, section 3.1). Only one microbial host of the TX core influent ARGs was identified in effluent: *bla_OXA_* in the chromosome of an *Aeromonadaceae* spp., the same family it was hosted by in influent (Fig. S4).

The CA influent had the most core influent ARGs detected (*n* = 36, Table 3). Most identified ARG-bacterial host relationships in influent were on plasmids (Table 3; Fig. S5). Additionally, numerous bacterial families were detected that hosted ARGs, including *Neisseriaceae* spp., *Enterobacteriaceae* spp., *Burkholderiaceae* spp., and *Bacteroidaceae* spp. (Fig. S5). *Neisseriaceae* spp. contained plasmids with *msr(E*) macrolide resistance, *sul1* sulfonamide-mediated resistance (34), and *tet(C*)-mediated tetracycline resistance (30). *Enterobacteriaceae* spp. contained the multidrug resistance genes *acrF* and *emrD* in the chromosome (Fig. S5) (35, 36). *Burkholderiaceae* spp. contained *msr(E*) and *sul1* on plasmids (Fig. S5). Finally, *Bacteroidaceae* spp. contained beta-lactamase *cfxA* resistance on a plasmid and *mef(B*) macrolide resistance in its chromosome (37, 38). While most ARGs were identified in bacterial plasmids in influent, in the AS most of the core influent ARGs were carried on bacterial chromosomes (Table 3; Fig. S5). In the AS, *sul1* was detected in each sample in the chromosome of *Competibacter phosphatis* (supplemental material, section 3.1) but was not detected in this family in the influent. *C. phosphatis* was detected only in the influent in the second week of sampling but without resistance genes. Of the CA core influent ARGs, only two bacterial hosts were identified in the effluent. *Moraxellaceae* spp. carried a plasmid with *msr(E*) and *Thiobacillaceae* spp. carried a plasmid with *sul1* (Fig. S5).

**Fig 4:**
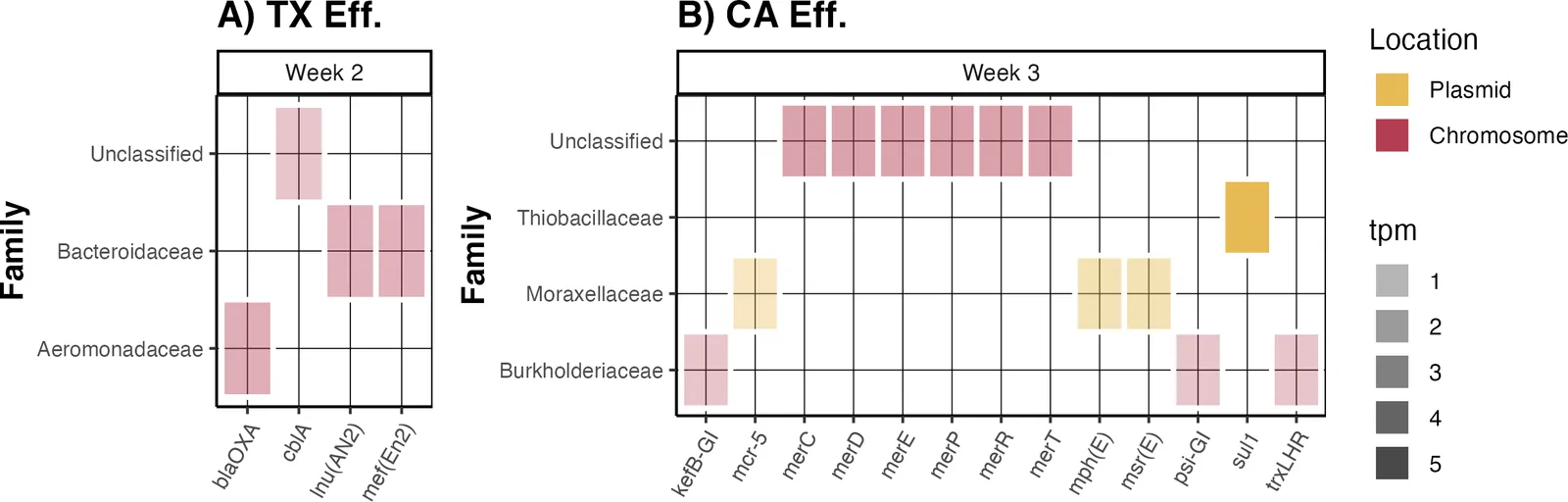
All ARG-host relationships detected by Hi-C contact mapping in effluent samples. Bacterial hosts were identified in (**A**) the Texas (TX) sample from week 2 and (**B**) the California (CA) sample from week 3. No other effluent samples from other sampling events had bacterial hosts for ARGs. Shading is representative of the TPM. Unclassified hosts were identified as plasmids and/or bacterial chromosomes crosslinked with resistance genes in the assemblies that did not meet sequencing quality criteria during classification.

Microbial hosts of all resistance genes in effluent were also assessed, with few hosts identified (Table 2 and Fig. 4). NC effluent had no ARGs with hosts, TX effluent had four ARGs hosted in chromosomes, and CA had 13 ARGs hosted in either plasmids or chromosomes (Table 2 and Fig. 4). All resistance genes detected in TX effluent conferred resistance to antibiotics, with one of the detected genes also in the core resistome (*bla_OXA_*; Fig. 4A). The *bla*_OXA_ gene was found in the chromosome of an *Aeromonadaceae* spp. in TX effluent. *Bacteroidaceae* spp. hosted *lnu(AN2*)-mediated lincomycin resistance and *mef(En2*)-mediated macrolide resistance (Fig. 4A) (39–41). CA effluent had four resistance genes on plasmids and nine in microbial chromosomes (Table 2 and Fig. 4B). Four of these effluent-hosted ARGs conferred drug resistance: *mcr-5, mph(E), msr(E),* and *sul1* (Fig. 4B). CA effluent contained *Moraxellaceae* spp. with colistin resistance (*mcr5*) and macrolide resistance [*mph(E*) and *msr(E*)] on plasmids (26, 27, 42, 43). Two ARGs detected in CA influent were detected in hosts from the same sampling week in the effluent: *msr(E*) and *sul1* (Fig. 4B; Fig. S5). The *sul1* gene is in an unclassified *Thiobacillus* species, and *msr(E*) is in a *Moraxellaceae* spp. in effluent (Fig. 4B).

### Priority resistant families

When considering only ARGs, 114 total bacterial hosts were identified by Hi-C contact maps (Table 4). More than 50% of the identified relationships involved four bacterial families: *Moraxellaceae* spp. (*n* = 22 ARGs), *Burkholderiaceae* spp. (*n* = 18 ARGs), *Aeromonadaceae* spp. (*n* = 13 ARGs), and *Neisseriaceae* spp. (*n* = 8 ARGs). These families also represented a high relative proportion of the families detected, with average relative abundances ranging from 6.3% to 14.6% (Table 4). While they were not detected in every sample, when present, they represented a relatively high proportion of the total bacterial community (Table 4). Although the bulk of resistance was found in the plasmids in these families from influent samples, there were resistant *Aeromonadaceae* spp. and *Moraxellaceae* spp. detected in the effluent (Table S5).

**TABLE 4:** ARGs conferring resistance to pharmaceuticals (*n* = 114) hosted by specific bacterial families across all sample types and geographic locations^*a*^

Family	No. of ARGs in family	% Total hosted ARGs	Mean rel. abun. (95% CI)	No. of samples collected with family detection (% total samples)
*Moraxellaceae*	22	19.3%	6.3% (−9.3% to 21.8%)	8 (33%)
*Burkholderiaceae*	18	15.8%	12.8% (−1.8% to 27.4%)	12 (50%)
*Aeromonadaceae*	13	11.4%	9.8% (−0.6% to 20.3%)	5 (21%)
*Neisseriaceae*	8	7.0%	14.6% (−0.7% to 29.9%)	5 (21%)
Everything else	53	46.5%		

^*a*^ Mean relative abundance (rel. abun.) is the average relative abundance of that family across each sample collected where that family was detected at >70% completion and <20% contamination.

## DISCUSSION

### Treatment stage affects location in microbial genome but not overall resistome

The resistome is highly stable across geographic location, with all resistance signatures in all samples collected from all three geographic locations dominated by beta-lactam, macrolide, and sulfonamide resistance (Table S3; Fig. 2A). These classes of antibiotics have been previously identified in environmental and clinical data as being under threat by growing resistance, with high detection of beta-lactam resistance in clinical samples (44) and a high prevalence of macrolide resistance in US wastewater (8). Furthermore, additional published research has found that the resistomes in influent (8) and AS (45) collected from the same continent are highly similar. Although antibiotic prescription data were not collected as part of this study, macrolide and penicillin (beta-lactam) antibiotics have regularly been identified as the most commonly prescribed antibiotics in the United States for over a decade (46, 47). It is therefore unsurprising that macrolide and beta-lactam resistance was dominant in this study. Although the resistomes across states were not statistically different, there was significant clustering by sample type identified by PERMANOVA in the resistome despite the use of different biological processes and final disinfection (Fig. S2; *P* < 0.001). The AS exhibited the least variability, potentially due to the maintenance of certain resistance profiles and microbes due to sludge retention in the system (48). Other metagenomic research has observed similar clustering by treatment stage even with different treatment processes implemented (48).

Despite the stability in the resistome, there is little community overlap between influent, AS, and effluent (Fig. 1). Influent microbes in NC and TX exhibited little similarity week to week, while CA influent was more stable, and AS from all three facilities was highly similar (Fig. 1). Stability in the resistome across geography, time, and treatment process despite changes in the underlying communities suggests that the resistome is not dependent on the microbial communities. Future analysis of the evidence of shared MGEs and plasmids to facilitate full characterization of AMR dynamics in WRFs is needed.

Although the overall resistome was highly stable across the WRFs tested here, resistance gene burden in shotgun data decreased from influent to effluent, with the bulk of the reduction occurring from influent to AS (Table 1). However, for sample weeks where the full bioinformatics pipeline was carried out in all three treatment processes, total resistance genes increased from AS to effluent (Table 1). This could indicate the existence of microbial reservoirs in the facility or be an effect of the sampling scheme, which did not take into consideration WRF hydraulic retention times (HRT), and sampling included grab and 24-hour composites. However, previous research does not find that HRT-informed sampling affected pathogen reduction estimates (49); less is known about the impact of sample timing on gene removal. Additionally, other studies have demonstrated the bulk of resistance reduction occurs between influent and AS (12, 48, 50), and that individual ARGs increased from influent to effluent (51), indicating the patterns observed here are likely not a function of HRT or the sampling scheme but represent true underlying patterns in the resistome. It is likely that observed patterns were not affected by the use of grab samples in AS and effluent, considering AS and effluent are a composite of many time points due to mixing upstream. However, for influent, it has been shown that composite samples better represent pathogen loading in wastewater due to diurnal use of bathroom facilities (52).

The location of resistance genes in microbial genomes is dependent on treatment step, as both total and core resistance genes are more likely to be in an intracellular plasmid in influent but in a microbial chromosome in AS (Table 2; Table S4). This relative decrease in the proportion of plasmid-based resistance genes from influent to AS has been observed by others (50). It was further expected that the influent resistome would be driven by plasmids, given that the gut microbiome is well captured in influent wastewater and the strong influence of plasmid-based resistance genes in the environment and clinical settings (9, 21, 53–56). While previous work can identify how chromosomal- and plasmid-based resistance genes change through treatment, they are limited by the inability to link plasmid-based genes to their host microbes, as shown here. Even though a majority of detected ARGs in this study did not have a microbial host identified (Table 2), this aligns with other work carried out at WRFs. P. Munk et al. (8) assessed AMR in influent wastewater using shotgun metagenomic sequencing and found microbial hosts for roughly 10% of identified ARGs. In the current study, 12.3% of ARGs were identified in a microbial host. While this is not a significant improvement on identifying microbial hosts from previous work, it is likely that sampling at WRFs will inherently result in reduced identification of microbial hosts. There are likely undescribed in-sewer effects resulting in microbial decay, particularly for human commensal and pathogenic bacteria (57). To fully resolve microbial hosts of the resistome, samples should be taken farther upstream to avoid any in-sewer decay. However, as the aims of the current study were to assess the resistome at WRFs, sampling upstream would not have answered study questions. The current study was able to identify both chromosomal resistance genes and successfully link genes on plasmids to microbial hosts due to the Hi-C sample preparation methods.

There were very few microbial hosts of resistance genes identified in effluent samples despite the total number of resistance genes increasing in effluent compared to AS (Tables 1 and 2, Fig. 4), highlighting both the success of the WRF treatment processes presented here at removing resistant bacteria and the difficulties of identifying microbial hosts in matrices with weak genetic signatures. While the facilities used different final disinfection processes (UV in NC, filtration and chlorination in TX, chlorination in CA), it is unclear if effluent host identification is an artifact of sequencing or a function of final disinfection. Existing research does not present a clear answer, as the identified effects of final disinfection on ARB are mixed (58), requiring future research on both ARB and ARGs. It is unlikely an artifact of sequencing, as extracellular DNA has been found previously to represent a larger fraction of total DNA in effluent compared to influent (59, 60). It is therefore unsurprising that there were few microbial hosts identified in WRF effluents, as much of this DNA was probably extracellular. However, the identification of ARGs in both microbial chromosomes and plasmids in effluent samples from TX and CA but not NC with Hi-C sequencing suggests that there are resistant bacteria escaping final disinfection with chlorine. While there are no conclusive results on the effects of final disinfection from the current study, there are likely quantitative differences unable to be elucidated here. As UV disinfection irreversibly crosslinks DNA while chlorination targets cellular structures (61, 62), it is likely that UV (used by NC) best prevents environmental dissemination of resistance genes that are available for bacterial acquisition. Furthermore, it is possible that there were more microbial hosts identified in CA effluent than TX effluent because TX uses filtration, which creates a physical barrier to prevent releasing both susceptible and resistant microbes (63). However, solids retention from filtration could result in higher ARB and ARG abundance in solids (63), which presents uniquely different dissemination and exposure pathways.

### Conserved WRF resistome provides resistance against heavy metals and antibiotics

The resistance genes that were shared among all samples tested, the core resistome, were representative of diverse resistance mechanisms, suggesting no single resistance mechanism is most protective for bacteria. First, four genes in the *mer* operon (*merE, merP, merR,* and *merT*) were identified in all samples that underwent the full bioinformatics pipeline (Table S4). Although mercury and other heavy metals were not measured here, heavy metals in WRFs have been found to be at high enough concentrations to increase plasmid conjugation frequency and drive resistance for various resistance genes (13, 64–67). There were also three commonly identified ARGs found in all samples analyzed (Table S4): *bla_OXA_*, which is one of the most commonly identified carbapenem resistance genes across sample types (68–70), and the macrolide resistance gene *msrE* and the tetracycline resistance gene *tetC,* which were both detected by P. Munk et al. (8) in 757 wastewater samples collected from 101 countries. Antibiotics were not analyzed here, but antibiotics are commonly detected in WRFs at levels that drive resistance (10). Future work should aim to quantify heavy metal and antibiotic concentrations to better understand drivers of AMR dynamics with Hi-C sequencing. Additionally, it is not always clear whether detection of resistance genes and ARB at WRFs represents the contributing population, is a function of in-sewer dynamics, or bacterial persistence in biofilms (57). Metagenomics sequencing, both presented here and elsewhere, is also often prohibitively expensive to sequence deeply enough to identify microbes at the species level, preventing deeper understanding of the microbial origins of resistance genes.

### Critical microbial families and species identified as ARG hosts

Although species-level annotation of microbial resistance gene hosts overall was limited, there were a few notable species-level detections. *A. caviae* with chromosomal *bla_OXA_* beta-lactam resistance was detected in NC and TX influent and TX effluent (Fig. 3; supplemental material, section 3.1). Beta-lactam-resistant *A. caviae* was previously isolated from advanced water purification facilities (71). Detection in influent and effluent from two of the WRFs sampled here suggests that this resistant bacterium is common in the population and can survive the wastewater treatment processes presented here and advanced water treatment processes previously reported. A chromosomal *msr(E*) encoded macrolide-resistant *A. johnsonii* bacterium was additionally detected in NC influent (Fig. 3; supplemental material, section 3.1). *A. johnsonii* is primarily a commensal bacterium but can cause opportunistic catheter-related bloodstream infections (72, 73). Although *A. johnsonii* has been proposed as a reservoir of ARGs (72), there is limited evidence it carries extensive macrolide resistance. This detection by Hi-C could represent either a new resistotype or further support the hypothesis that it is an ARG reservoir. Bacterial species involved in the nitrogen cycle, *Nitrospira defluvii* (*Nitrospiraceae* family) and *Comamonas denitrificans* (*Burkholderiaceae* family), were identified with plasmid-based *mer* mercury resistance and *msr(E*) macrolide resistance in NC influent and AS (Fig. 3; supplemental material, section 3.1), indicating resistome dynamics are not limited to clinical bacteria in these facilities. Future work should aim to incorporate plasmid–microbe relationships identified by Hi-C into existing resistome risk frameworks such as those presented by Majeed et al. (7) and Zhang et al. (74). As mobile plasmids are able to be linked to microbial hosts with Hi-C sequencing, additional risk categories are likely needed.

There were additionally several microbial-ARG host relationships identified in AS and effluent that were not detected in influent. Each AS sample collected in CA contained *C. phosphatis* with *sul1* chromosomal sulfonamide resistance (Fig. S5 and supplemental material, section 3.1), despite a lack of this relationship in influent. *C. phosphatis* is a Candidatus species proposed to be a glycogen-accumulating organism that is typically involved with anaerobic accumulation of glycogen in WRFs carrying out enhanced biological phosphate removal (EBPR), although metagenomics suggest these bacteria can also aerobically utilize lactate in alternative metabolic pathways (75, 76). While *C. phosphatis* is regularly detected in EBPR facilities (75, 76), it has not been regularly detected in aerobic activated sludge systems like the process used in the CA WRF (77). This indicates that the enduring *C. phosphatis* detected here is likely utilizing an alternative aerobic metabolic pathway and that *sul1* aids in its persistence. While there are likely other sources of *sul1* in the WRF, recurrent detection suggests persistence of this ARG-microbial relationship independent of influent bacteria.

Among the resistance genes detected in TX effluent, only *bla_OXA_* was in the same microbial species (*Aeromonas caviae*) in effluent and influent (Fig. 3B and 4A; supplemental material, section 3.1). Further in CA, the only two ARGs identified with microbial hosts in effluent that were also detected in influent [*sul1* and *msr(E*)] were in plasmids in different microbial hosts in effluent than in influent. This could suggest either sporadic detection of resistant bacteria throughout the WRF with Hi-C or be representative of AMR dynamics in the facilities. There is no obvious pattern in the resistant bacteria that escape final disinfection, as there are ARGs conferring resistance to both heavy metals (*mer* operon) and antibiotics including beta-lactams (*bla_OXA_, cblA*), lincosamides [*lnu(AN2*)], macrolides [*mef(En2), mph(E),* and *msr(E*)], colistin (*mcr-5*), and sulfonamides (*sul1*) carried on both plasmids and in bacterial chromosomes. These ARGs prevent both bactericidal and bacteriostatic effects. However, in both TX and CA, all ARGs detected in the same family were also detected in the same MAG, suggesting there is a benefit to carrying multiple resistance genes through treatment. Bench-scale experiments should be carried out to understand the protective effects of carrying multiple resistance genes against different types of final disinfection. Even though species-level identification was limited, as shown here, Hi-C can be used to better understand the resistome without isolating and culturing resistant bacteria.

A majority of ARGs for pharmaceuticals were hosted by four bacterial families: *Moraxellaceae* spp., *Burkholderiaceae* spp., *Aeromonadaceae* spp., and *Neisseriaceae* spp. (Table 4). Furthermore, a majority of ARGs with bacterial hosts were detected in plasmids (Table S5), suggesting the risk for HGT is high. The Hi-C contact maps indicate that these four bacterial families are AMR reservoirs. Both *Moraxellaceae* spp. and *Aeromonadaceae* spp. were previously identified as AMR reservoirs in wastewater treatment systems (20, 78). Furthermore, the presence of resistant and intact *Aeromonadaceae* spp. and *Moraxellaceae* spp. in WRF effluents (Fig. 4; Table S5) emphasizes the need to understand drivers of these bacterial families to reduce the probability of environmental AMR exposure and HGT of the genes they carry.

### Study limitations

There are several limitations of the current study. First, Hi-C has the same limitations as shotgun sequencing in that metagenomic annotation is dependent on the databases utilized and sequencing quality and depth affect final results. Therefore, there are likely unidentified relationships in these samples that would benefit from more targeted single-cell sequencing techniques like epicPCR and long-read sequencing with unique molecular identifiers (19, 79). Additionally, the sample size is relatively small and the time covered is too short to identify temporal changes in the resistome. Future work should aim to collect seasonal data to better understand temporal changes. Although we were able to show that resistance genes are less likely to be in a microbial host through treatment, we were not able to identify significant differential final disinfection effects at the three WRFs. Method development for effluent samples is likely needed given the low concentrations of DNA in these samples, as well as enrolling more facilities in the study. Future research should additionally investigate disinfection of secondary effluent from a single facility with different methods to more quantitatively and mechanistically understand the effect of disinfection on the resistome microbial host range. Despite these study limitations, the novelty of proximity ligation techniques helps advance understanding of the resistome in complex environmental matrices.

### Conclusions

The current study identified numerous ARG-microbial relationships and potential reservoirs of AMR in water reclamation facilities in three geographically distinct areas. *Moraxellaceae* spp., *Burkholderiaceae* spp., *Aeromonadaceae* spp., and *Neisseriaceae* spp. were together responsible for more than 50% of microbial-ARG host relationships. Resistance genes were detected in both chromosomes and plasmids in numerous microbial families across wastewater treatment, suggesting Hi-C elucidates resistance gene hosts across bacterial families. Furthermore, the location of ARGs in the microbial genome was dependent on the treatment step, as ARGs were more likely to be in microbial plasmids in influent and in microbial chromosomes in AS. While total ARGs decreased from influent to effluent, there were increases in ARGs from AS to effluent in individual sample weeks, and resistant bacteria were detected in effluent. Overall, more research into the specific drivers of ARG transfer in WRFs is needed to inform risk assessments. Hi-C contact mapping is a powerful shotgun sequencing tool to identify ARG hosts in complex environmental matrices. Hi-C and other proximity ligation sequencing methods are the only nonspecific methods to definitively link MGEs with bacterial hosts by physically crosslinking plasmids with bacterial chromosomes before cell lysis. The results presented here advance understanding of the resistome in WRFs, particularly for resistance genes carried on plasmids.

## MATERIALS AND METHODS

### Sample collection and concentration

Samples were collected weekly during the same weeks from three WRFs in CA, TX, and NC. The WRF in CA treats over 400 million gallons a day of wastewater from an urban population with a mix of domestic and industrial waste streams. The TX WRF primarily treats residential wastewater with flows over 80 MGD. The NC WRF is the smallest of the three, treating approximately 10 MGD of primarily residential wastewater with some industrial inputs. All three WRFs discharge the effluent into local surface waters. Primary influent was either grab-sampled in the morning (NC) or collected as a 24-hour composite sample (CA and TX). Activated sludge (AS) was grab-sampled from all three WRFs. Effluent was collected after disinfection with either UV (NC) or chlorine (CA and TX) via grab sampling (NC and TX) or a 24-hour composite (CA). Sample collection volumes and dates are in Table S6. Samples collected in TX and NC were shipped overnight with refrigeration to the ReWater Center at the University of Southern California (USC). All samples were then stored at 4°C until processing.

Primary influent (50 mL) and AS (2.0 mL) from all three WRFs were centrifuged at 12,000 × *g* and 4°C for 10 minutes (21). The supernatant was then removed, and the pellets were stored at 4°C. Effluent (500 mL) was centrifuged at 3,500 × *g* and 4°C for 30 minutes in 50 mL conical centrifuge tubes. The supernatant was removed, and pellets were combined. The combined pellets were then recentrifuged and the supernatant removed for a single pellet from 500 mL equivalent effluent volume. This pellet was stored at 4°C. All processing for all three sample types was carried out in duplicate, with one replicate used for Hi-C crosslinking and the other for DNA extraction and shotgun sequencing.

### Hi-C preparation

Hi-C crosslinking was carried out within 1 week of collection and 24 hours of receiving the samples at USC. One sample replicate from each location, sample type, and sampling date was processed using the ProxiMeta Hi-C Kit following protocol v4.5.4 (Phase Genomics, Seattle, WA, USA) with the following alterations to crosslinking to account for solids content. Samples were crosslinked with 10 mL of a chilled 1× PBS with 1% formaldehyde solution for 10 minutes at room temperature. Crosslinking was quenched with 527 μL of 2.5 M glycine for 5 minutes. Samples were then transferred to ice for 10 minutes with periodic agitation. After chilling, samples were centrifuged at 3,500 × *g* and 4°C for 10 minutes. The supernatant was discarded, and the pellets were resuspended in 10 mL of chilled 1× PBS. Resuspended samples were centrifuged again at 3,500 × *g* and 4°C for 10 minutes. The supernatant was discarded, and the pellets were resuspended in 1.0 mL of chilled 1× PBS. Samples were centrifuged at 800 × *g* and 4°C for 5 minutes, supernatant discarded, and crosslinked pellets stored at −80°C until further processing (80). After crosslinking, processing followed the protocol as written.

### DNA extraction

DNA was automatically extracted from remaining pellet replicates using the Maxwell Blood DNA Kit using the Maxwell RSC 48 instrument (Promega Corp., Madison, WI, USA). Cell lysis was modified to account for additional solids in wastewater samples. Briefly, 300 μL of the included lysis buffer and 0.5 g of sterilized zirconia beads (BioSpec Products Inc, Bartlesville, OK) were added to the pellets. Samples underwent bead beating at 2,400 RPM for 2 minutes using a Mini-BeadBeater 96 (BioSpec Products Inc, Bartlesville, OK), followed by centrifugation at 15,000 × *g* for 5 minutes. A total of 200 μL of supernatant was transferred to a sterile 1.5 mL microcentrifuge tube. In total, 200 μL of lysis buffer was added to the bead tube. The whole process was repeated twice for three total rounds, resulting in 600 μL total of cell lysate. Proteinase K was then added to the cell lysate (60 μL) and mixed by vortexing. Cell lysate was incubated at 56°C for 30 minutes. The extraction protocol then proceeded as written with final elution in 40 μL of nuclease-free water.

### Sequencing and bioinformatics analysis

DNA extracts were sent to the Oklahoma Medical Research Foundation Clinical Genomics Core (Oklahoma City, OK) for library preparation and shotgun sequencing. Shotgun libraries were prepared using a Watchmaker DNA Library Prep Kit (Watchmaker Genomics Boulder, CO). Indexed Hi-C sample libraries were sent to the Duke Sequencing and Genomic Technologies Core Facility (Durham, NC) for sample pooling and shotgun sequencing. Both the shotgun and Hi-C libraries were sequenced at a depth of 100 million reads/sample using one lane of an Illumina NovaSeq X Plus (Illumina San Diego, CA).

Shotgun sequencing libraries were assembled utilizing the USC Center for Advanced Research Computing’s high-performance computing systems. Initial quality control analysis of the fastq files was performed using FastQC (81). Low-quality reads and adapters were filtered and trimmed using bbDuk (82). Contigs were assembled using metaSPADES (83). Mean contig length for all samples that underwent Hi-C deconvolution was obtained using the quast.py command from QUAST (v5.0.3) and is in Table S1 (84, 85). The commands with all flags are in the supplemental material, Appendix B.

Hi-C deconvolution, binning, and annotation were performed at Phase Genomics (Seattle, Washington, USA) using the ProxiMeta platform following similar methods as described in M. O. Press et al. (86) and A. Risely et al. (21). In brief, Hi-C reads were aligned to the metagenomic assemblies with the –5SP flag using BWA-MEM (87), the alignments were filtered using the –F 2304 flag with SAMtools (88), and assemblies were binned using the ProxiMeta platform (86, 89). In contrast with binning traditional shotgun sequencing data, Hi-C binning incorporates the crosslinked DNA to bin the assemblies into genomes, plasmids, and viruses rather than relying solely on sequence similarity (86). Average coverage of the Hi-C-generated bins to the trimmed shotgun sequencing reads was calculated using the CoverM genome command with the -p flag using BWA-MEM and the -m flag using TPM (90).

The Genome Taxonomy Database Toolkit (GTDB-tk, v2.4.0) and database v220 were used to classify bin taxonomy (91). Plasmid contigs were identified by blastn of all contigs using the PLSD database (v2020_06_29), and the Hi-C data were used to bin plasmids into pMAGs (92, 93). AMR genes were annotated from the metagenomic assemblies using AMRFinderPlus (v3.10.5). The gene annotations were cross-referenced with all MAGs and host associations and were assigned to their origin by using a binary matrix. MAGs were generated by filtering assembled bins for >70% completion and <20% contamination using the dplyr R package (94). Three effluent samples (one from NC and two from CA) were not able to undergo the complete Hi-C pipeline because there was not sufficient mapping of the Hi-C reads to the assemblies, and these samples were removed from subsequent analysis.

### Data management and statistical analysis

All statistical analysis and data manipulation were carried out using R (95), RStudio (96), and affiliated packages. Data were imported into RStudio using readr (97). Data were manipulated using the dplyr and tidyverse packages (94, 98). Plots were generated using ggplot2 (99), ggh4x (100), scico (101), pals (102), ggpubr (103), cowplot (104), and ggbiplot (105). Relative abundance (Rel. abun.) of the MAGs, antibiotic resistance genes, and total resistance to antibiotic classes was calculated by adjusting the TPM reads to the relative fraction for each target as follows (equation 1):


(1) $$Rel.\;abun.\;=\;\frac{\sum_{a=1}^{n}a}{\sum_{b=1}^{n}b}\times 100$$

where *a* is TPM for a single MAG, antibiotic resistance gene, or antibiotic class and *b* is TPM for all MAGs or antibiotic resistance genes.

Although transcripts were not analyzed in this study, TPM is a metric that normalizes shotgun sequencing data to the read length and sequencing depth to allow for comparability between samples and is comparable to relative abundance. Principal component analysis (PCA) with centering and scaling following Hellinger transformation of the data were carried out using the prcomp function in the R Stats Package, the labdsv package (106), and the factoextra package (107). Microbial community and AMR profile clustering following Hellinger transformation was assessed with a PERMANOVA using the adonis2 command of the vegan package (108).

## Data Availability

Raw sequences are deposited in the National Center for Biotechnology Information’s Sequence Read Archive database at project number PRJNA1295499.

## SUPPLEMENTAL MATERIAL

Supplemental material aem.00701-26-s0001.pdf Fig. S1 to S5, Tables S1 to S6, and supplemental methods and data sets.

Graphical abstract aem.00701-26-s0002.png Visual depiction of the study highlights.

ASM does not own the copyrights to Supplemental Material that may be linked to, or accessed through, an article. The authors have granted ASM a non-exclusive, world-wide license to publish the Supplemental Material files. Please contact the corresponding author directly for reuse.

## References

[1] Naghavi M, Vollset SE, Ikuta KS, Swetschinski LR, Gray AP, Wool EE, Robles Aguilar G, Mestrovic T, Smith G, Han C, et al.. 2024. Global burden of bacterial antimicrobial resistance 1990–2021: a systematic analysis with forecasts to 2050. The Lancet 404:1199–1226. doi:10.1016/S0140-6736(24)01867-1 PMC11718157 39299261

[2] Liu Y-Y, Wang Y, Walsh TR, Yi L-X, Zhang R, Spencer J, Doi Y, Tian G, Dong B, Huang X, et al.. 2016. Emergence of plasmid-mediated colistin resistance mechanism MCR-1 in animals and human beings in China: a microbiological and molecular biological study. Lancet Infect Dis 16:161–168. doi:10.1016/S1473-3099(15)00424-7 26603172

[3] Kumarasamy KK, Toleman MA, Walsh TR, Bagaria J, Butt F, Balakrishnan R, Chaudhary U, Doumith M, Giske CG, Irfan S, et al.. 2010. Emergence of a new antibiotic resistance mechanism in India, Pakistan, and the UK: a molecular, biological, and epidemiological study. Lancet Infect Dis 10:597–602. doi:10.1016/S1473-3099(10)70143-2 20705517 PMC2933358

[4] Liguori K, Keenum I, Davis BC, Calarco J, Milligan E, Harwood VJ, Pruden A. 2022. Antimicrobial resistance monitoring of water environments: a framework for standardized methods and quality control. Environ Sci Technol 56:9149–9160. doi:10.1021/acs.est.1c08918 35732277 PMC9261269

[5] Johnson J. 2023. CDC greatly expands number of infectious disease targets for National wastewater surveillance. GenomeWeb. Available from: https://www.genomeweb.com/pcr/cdc-greatly-expands-number-infectious-disease-targets-national-wastewater-surveillance

[6] Pruden A, Vikesland PJ, Davis BC, de Roda Husman AM. 2021. Seizing the moment: now is the time for integrated global surveillance of antimicrobial resistance in wastewater environments. Curr Opin Microbiol 64:91–99. doi:10.1016/j.mib.2021.09.013 34655936

[7] Majeed HJ, Riquelme MV, Davis BC, Gupta S, Angeles L, Aga DS, Garner E, Pruden A, Vikesland PJ. 2021. Evaluation of metagenomic-enabled antibiotic resistance surveillance at a conventional wastewater treatment plant. Front Microbiol 12:657954. doi:10.3389/fmicb.2021.657954 34054755 PMC8155483

[8] Munk P, Brinch C, Møller FD, Petersen TN, Hendriksen RS, Seyfarth AM, Kjeldgaard JS, Svendsen CA, van Bunnik B, Berglund F, et al.. 2022. Genomic analysis of sewage from 101 countries reveals global landscape of antimicrobial resistance. Nat Commun 13:7251. doi:10.1038/s41467-022-34312-7 36456547 PMC9715550

[9] Larsson DGJ, Flach C-F. 2022. Antibiotic resistance in the environment. Nat Rev Microbiol 20:257–269. doi:10.1038/s41579-021-00649-x 34737424 PMC8567979

[10] Michael I, Rizzo L, McArdell CS, Manaia CM, Merlin C, Schwartz T, Dagot C, Fatta-Kassinos D. 2013. Urban wastewater treatment plants as hotspots for the release of antibiotics in the environment: a review. Water Res 47:957–995. doi:10.1016/j.watres.2012.11.027 23266388

[11] Rizzo L, Manaia C, Merlin C, Schwartz T, Dagot C, Ploy MC, Michael I, Fatta-Kassinos D. 2013. Urban wastewater treatment plants as hotspots for antibiotic resistant bacteria and genes spread into the environment: a review. Sci Total Environ 447:345–360. doi:10.1016/j.scitotenv.2013.01.032 23396083

[12] Keenum I, Calarco J, Majeed H, Hager-Soto EE, Bott C, Garner E, Harwood VJ, Pruden A. 2024. To what extent do water reuse treatments reduce antibiotic resistance indicators? A comparison of two full-scale systems. Water Res 254:121425. doi:10.1016/j.watres.2024.121425 38492480

[13] Nguyen AQ, Vu HP, Nguyen LN, Wang Q, Djordjevic SP, Donner E, Yin H, Nghiem LD. 2021. Monitoring antibiotic resistance genes in wastewater treatment: Current strategies and future challenges. Sci Total Environ 783:146964. doi:10.1016/j.scitotenv.2021.146964 33866168

[14] Quintela-Baluja M, Abouelnaga M, Romalde J, Su J-Q, Yu Y, Gomez-Lopez M, Smets B, Zhu Y-G, Graham DW. 2019. Spatial ecology of a wastewater network defines the antibiotic resistance genes in downstream receiving waters. Water Res 162:347–357. doi:10.1016/j.watres.2019.06.075 31295654 PMC6650630

[15] Makowska N, Koczura R, Mokracka J. 2016. Class 1 integrase, sulfonamide and tetracycline resistance genes in wastewater treatment plant and surface water. Chemosphere 144:1665–1673. doi:10.1016/j.chemosphere.2015.10.044 26519797

[16] Berglund B, Fick J, Lindgren PE. 2015. Urban wastewater effluent increases antibiotic resistance gene concentrations in a receiving northern European river. Environ Toxicol Chem 34:192–196. doi:10.1002/etc.2784 25331227

[17] Hiller CX, Hübner U, Fajnorova S, Schwartz T, Drewes JE. 2019. Antibiotic microbial resistance (AMR) removal efficiencies by conventional and advanced wastewater treatment processes: a review. Sci Total Environ 685:596–608. doi:10.1016/j.scitotenv.2019.05.315 31195321

[18] Aarestrup FM, Woolhouse MEJ. 2020. Using sewage for surveillance of antimicrobial resistance. Science 367:630–632. doi:10.1126/science.aba3432 32029617

[19] Roman VL, Merlin C, Virta MPJ, Bellanger X. 2021. EpicPCR 2.0: technical and methodological improvement of a cutting-edge single-cell genomic approach. Microorganisms 9:1649. doi:10.3390/microorganisms9081649 34442728 PMC8399275

[20] Stalder T, Press MO, Sullivan S, Liachko I, Top EM. 2019. Linking the resistome and plasmidome to the microbiome. ISME J 13:2437–2446. doi:10.1038/s41396-019-0446-4 31147603 PMC6776055

[21] Risely A, Newbury A, Stalder T, Simmons BI, Top EM, Buckling A, Sanders D. 2024. Host- plasmid network structure in wastewater is linked to antimicrobial resistance genes. Nat Commun 15:555. doi:10.1038/s41467-024-44827-w 38228585 PMC10791616

[22] McCorison CB, Kim T, Donato JJ, LaPara TM. 2025. Proximity-ligation metagenomic sequence analysis reveals that the antibiotic resistome makes significant transitions during municipal wastewater treatment. Environ Microbiol 27:e70036. doi:10.1111/1462-2920.70036 39797441 PMC11724201

[23] Osborn AM, Bruce KD, Strike P, Ritchie DA. 1997. Distribution, diversity and evolution of the bacterial mercury resistance (mer) operon. FEMS Microbiol Rev 19:239–262. doi:10.1111/j.1574-6976.1997.tb00300.x 9167257

[24] Boyd ES, Barkay T. 2012. The mercury resistance operon: from an origin in a geothermal environment to an efficient detoxification machine. Front Microbiol 3:349. doi:10.3389/fmicb.2012.00349 23087676 PMC3466566

[25] Antunes NT, Fisher JF. 2014. Acquired class D β-lactamases. Antibiotics (Basel) 3:398–434. doi:10.3390/antibiotics3030398 27025753 PMC4790369

[26] Ero R, Kumar V, Su W, Gao Y-G. 2019. Ribosome protection by ABC-F proteins-molecular mechanism and potential drug design. Protein Sci 28:684–693. doi:10.1002/pro.3589 30746819 PMC6423996

[27] Chen Q, Lu W, Zhou D, Zheng G, Liu H, Qian C, Zhou W, Lu J, Ni L, Bao Q, Li A, Xu T, Xu H. 2020. Characterization of two macrolide resistance-related genes in multidrug-resistant *Pseudomonas aeruginosa* isolates. Pol J Microbiol 69:349–356. doi:10.33073/pjm-2020-038 33574864 PMC7810118

[28] Chopra I, Roberts M. 2001. Tetracycline antibiotics: mode of action, applications, molecular biology, and epidemiology of bacterial resistance. Microbiol Mol Biol Rev 65:232–260. doi:10.1128/MMBR.65.2.232-260.2001 11381101 PMC99026

[29] Roberts MC. 2005. Update on acquired tetracycline resistance genes. FEMS Microbiol Lett 245:195–203. doi:10.1016/j.femsle.2005.02.034 15837373

[30] Peden KWC. 1983. Revised sequence of the tetracycline-resistance gene of pBR322. Gene 22:277–280. doi:10.1016/0378-1119(83)90112-9 6307828

[31] Alcock BP, Huynh W, Chalil R, Smith KW, Raphenya AR, Wlodarski MA, Edalatmand A, Petkau A, Syed SA, Tsang KK, et al.. 2023. CARD 2023: expanded curation, support for machine learning, and resistome prediction at the Comprehensive Antibiotic Resistance Database. Nucleic Acids Res 51:D690–D699. doi:10.1093/nar/gkac920 36263822 PMC9825576

[32] Szczepanowski R, Krahn I, Bohn N, Pühler A, Schlüter A. 2007. Novel macrolide resistance module carried by the IncP-1beta resistance plasmid pRSB111, isolated from a wastewater treatment plant. Antimicrob Agents Chemother 51:673–678. doi:10.1128/AAC.00802-06 17101677 PMC1797757

[33] Nonaka L, Maruyama F, Suzuki S, Masuda M. 2015. Novel macrolide-resistance genes, mef(C) and mph(G), carried by plasmids from *Vibrio* and *Photobacterium* isolated from sediment and seawater of a coastal aquaculture site. Lett Appl Microbiol 61:1–6. doi:10.1111/lam.12414 25765542

[34] Sköld O. 2001. Resistance to trimethoprim and sulfonamides. Vet Res 32:261–273. doi:10.1051/vetres:2001123 11432417

[35] Lau SY, Zgurskaya HI. 2005. Cell division defects in *Escherichia coli* deficient in the multidrug efflux transporter AcrEF-TolC. J Bacteriol 187:7815–7825. doi:10.1128/JB.187.22.7815-7825.2005 16267305 PMC1280316

[36] Yin Y, He X, Szewczyk P, Nguyen T, Chang G. 2006. Structure of the multidrug transporter EmrD from *Escherichia coli*. Science 312:741–744. doi:10.1126/science.1125629 16675700 PMC3152482

[37] Parker AC, Smith CJ. 1993. Genetic and biochemical analysis of a novel Ambler class A beta-lactamase responsible for cefoxitin resistance in *Bacteroides* species. Antimicrob Agents Chemother 37:1028–1036. doi:10.1128/AAC.37.5.1028 8517690 PMC187887

[38] Liu J, Keelan P, Bennett PM, Enne VI. 2009. Characterization of a novel macrolide efflux gene, mef(B), found linked to sul3 in porcine *Escherichia coli*. J Antimicrob Chemother 63:423–426. doi:10.1093/jac/dkn523 19131424

[39] Rivera-Mendoza D, Martínez-Flores I, Santamaría RI, Lozano L, Bustamante VH, Pérez-Morales D. 2020. Genomic analysis reveals the genetic determinants associated with antibiotic resistance in the zoonotic pathogen *Campylobacter* spp. distributed globally. Front Microbiol 11:513070. doi:10.3389/fmicb.2020.513070 33042043 PMC7518152

[40] Sydenham TV, Sóki J, Hasman H, Wang M, Justesen US. 2015. Identification of antimicrobial resistance genes in multidrug-resistant clinical *Bacteroides fragilis* isolates by whole genome shotgun sequencing. Anaerobe 31:59–64. doi:10.1016/j.anaerobe.2014.10.009 25464141

[41] Wang J, Shoemaker NB, Wang GR, Salyers AA. 2000. Characterization of a *Bacteroides* mobilizable transposon, NBU2, which carries a functional lincomycin resistance gene. J Bacteriol 182:3559–3571. doi:10.1128/JB.182.12.3559-3571.2000 10852890 PMC101958

[42] Schlüter A, Szczepanowski R, Pühler A, Top EM. 2007. Genomics of IncP-1 antibiotic resistance plasmids isolated from wastewater treatment plants provides evidence for a widely accessible drug resistance gene pool. FEMS Microbiol Rev 31:449–477. doi:10.1111/j.1574-6976.2007.00074.x 17553065

[43] Borowiak M, Fischer J, Hammerl JA, Hendriksen RS, Szabo I, Malorny B. 2017. Identification of a novel transposon-associated phosphoethanolamine transferase gene, mcr-5, conferring colistin resistance in d-tartrate fermenting *Salmonella enterica* subsp. *enterica* serovar paratyphi B. J Antimicrob Chemother 72:3317–3324. doi:10.1093/jac/dkx327 28962028

[44] Centers for Disease Control and Prevention. 2024. Antimicrobial resistance threats in the United States, 2021 - 2022

[45] Zhu C, Wu L, Ning D, Tian R, Gao S, Zhang B, Zhao J, Zhang Y, Xiao N, Wang Y, et al.. 2025. Global diversity and distribution of antibiotic resistance genes in human wastewater treatment systems. Nat Commun 16:4006. doi:10.1038/s41467-025-59019-3 40301344 PMC12041579

[46] Hicks LA, Bartoces MG, Roberts RM, Suda KJ, Hunkler RJ, Taylor TH, Schrag SJ. 2015. US outpatient antibiotic prescribing variation according to geography, patient population, and provider specialty in 2011. Clin Infect Dis 60:1308–1316. doi:10.1093/cid/civ076 25747410

[47] Centers for Disease Control and Prevention. 2024. Outpatient antibiotic use: retail pharmacy prescription data

[48] Honda R, Matsuura N, Sorn S, Asakura S, Morinaga Y, Van Huy T, Sabar MA, Masakke Y, Hara-Yamamura H, Watanabe T. 2023. Transition of antimicrobial resistome in wastewater treatment plants: impact of process configuration, geographical location and season. npj Clean Water 6:46. doi:10.1038/s41545-023-00261-x

[49] Sidhu JPS, Ahmed W, Palmer A, Smith K, Hodgers L, Toze S. 2017. Optimization of sampling strategy to determine pathogen removal efficacy of activated sludge treatment plant. Environ Sci Pollut Res Int 24:19001–19010. doi:10.1007/s11356-017-9557-5 28656581

[50] Dai D, Brown C, Bürgmann H, Larsson DGJ, Nambi I, Zhang T, Flach C-F, Pruden A, Vikesland PJ. 2022. Long-read metagenomic sequencing reveals shifts in associations of antibiotic resistance genes with mobile genetic elements from sewage to activated sludge. Microbiome 10:20. doi:10.1186/s40168-021-01216-5 35093160 PMC8801152

[51] Garner E, Maile-Moskowitz A, Angeles LF, Flach C-F, Aga DS, Nambi I, Larsson DGJ, Bürgmann H, Zhang T, Vikesland PJ, et al.. 2024. Metagenomic profiling of internationally sourced sewage influents and effluents yields insight into selecting targets for antibiotic resistance monitoring. Environ Sci Technol 58:16547–16559. doi:10.1021/acs.est.4c03726 39229966 PMC11411718

[52] Bivins A, North D, Wu Z, Shaffer M, Ahmed W, Bibby K. 2021. Within- and between-day variability of SARS-CoV-2 RNA in municipal wastewater during periods of varying COVID-19 prevalence and positivity. ACS EST Water 1:2097–2108. doi:10.1021/acsestwater.1c00178

[53] Castañeda-Barba S, Top EM, Stalder T. 2024. Plasmids, a molecular cornerstone of antimicrobial resistance in the One Health era. Nat Rev Microbiol 22:18–32. doi:10.1038/s41579-023-00926-x 37430173 PMC12440250

[54] Bennett PM. 2008. Plasmid encoded antibiotic resistance: acquisition and transfer of antibiotic resistance genes in bacteria. British J Pharmacology 153:MID–18193080. doi:10.1038/sj.bjp.0707607 PMC2268074 18193080

[55] San Millan A. 2018. Evolution of plasmid-mediated antibiotic resistance in the clinical context. Trends Microbiol 26:978–985. doi:10.1016/j.tim.2018.06.007 30049587

[56] Newton RJ, McLellan SL, Dila DK, Vineis JH, Morrison HG, Eren AM, Sogin ML. 2015. Sewage reflects the microbiomes of human populations. mBio 6:e02574. doi:10.1128/mBio.02574-14 25714718 PMC4358014

[57] Philo SE, De León KB, Noble RT, Zhou NA, Alghafri R, Bar-Or I, Darling A, D’Souza N, Hachimi O, Kaya D, et al.. 2024. Wastewater surveillance for bacterial targets: current challenges and future goals. Appl Environ Microbiol 90:e0142823. doi:10.1128/aem.01428-23 38099657 PMC10807411

[58] Bouki C, Venieri D, Diamadopoulos E. 2013. Detection and fate of antibiotic resistant bacteria in wastewater treatment plants: a review. Ecotoxicol Environ Saf 91:1–9. doi:10.1016/j.ecoenv.2013.01.016 23414720

[59] Tamai S, Okuno M, Ogura Y, Suzuki Y. 2025. Genetic diversity of dissolved free extracellular DNA compared to intracellular DNA in wastewater treatment plants. Sci Total Environ 970:178989. doi:10.1016/j.scitotenv.2025.178989 40048953

[60] Savin M, Hammerl JA, Hassa J, Hembach N, Kalinowski J, Schwartz T, Droop F, Mutters NT. 2023. Free-floating extracellular DNA (exDNA) in different wastewaters: status quo on exDNA-associated antimicrobial resistance genes. Environ Pollut 337:122560. doi:10.1016/j.envpol.2023.122560 37716694

[61] Cutler TD, Zimmerman JJ. 2011. Ultraviolet irradiation and the mechanisms underlying its inactivation of infectious agents. Anim Health Res Rev 12:15–23. doi:10.1017/S1466252311000016 21676338

[62] Venkobachar C, Iyengar L, Prabhakara Rao AVS. 1977. Mechanism of disinfection: Effect of chlorine on cell membrane functions. Water Res 11:727–729. doi:10.1016/0043-1354(77)90114-2

[63] Bairán G, Rebollar-Pérez G, Chávez-Bravo E, Torres E. 2020. Treatment processes for microbial resistance mitigation: the technological contribution to tackle the problem of antibiotic resistance. Int J Environ Res Public Health 17:8866. doi:10.3390/ijerph17238866 33260585 PMC7730199

[64] Suess E, Berg M, Bouchet S, Cayo L, Hug SJ, Kaegi R, Voegelin A, Winkel LHE, Tessier E, Amouroux D, et al.. 2020. Mercury loads and fluxes from wastewater: a nationwide survey in Switzerland. Water Res 175:115708. doi:10.1016/j.watres.2020.115708 32220669

[65] Feng J, Burke IT, Chen X, Stewart DI. 2023. Assessing metal contamination and speciation in sewage sludge: implications for soil application and environmental risk. Rev Environ Sci Biotechnol 22:1037–1058. doi:10.1007/s11157-023-09675-y

[66] Li W, Zhang W-G, Zhang M-S, Lei Z-F, Li P-F, Ma Y, Gao Y. 2022. Environmentally relevant concentrations of mercury facilitate the horizontal transfer of plasmid-mediated antibiotic resistance genes. Science of The Total Environment 852:158272. doi:10.1016/j.scitotenv.2022.158272 36028018

[67] Jones V, Gardner M, Ellor B. 2014. Concentrations of trace substances in sewage sludge from 28 wastewater treatment works in the UK. Chemosphere 111:478–484. doi:10.1016/j.chemosphere.2014.04.025 24997955

[68] Zhang A, Call DR, Besser TE, Liu J, Jones L, Wang H, Davis MA. 2019. β-lactam resistance genes in bacteriophage and bacterial DNA from wastewater, river water, and irrigation water in Washington State. Water Res 161:335–340. doi:10.1016/j.watres.2019.06.026 31212239

[69] Hultman J, Tamminen M, Pärnänen K, Cairns J, Karkman A, Virta M. 2018. Host range of antibiotic resistance genes in wastewater treatment plant influent and effluent. FEMS Microbiol Ecol 94. doi:10.1093/femsec/fiy038 PMC5939699 29514229

[70] Lee Y-L, Wang W-Y, Ko W-C, Hsueh P-R. 2024. Global epidemiology and antimicrobial resistance of Enterobacterales harbouring genes encoding OXA-48-like carbapenemases: insights from the results of the Antimicrobial Testing Leadership and Surveillance (ATLAS) programme 2018-2021. J Antimicrob Chemother 79:1581–1589. doi:10.1093/jac/dkae140 38758189

[71] Labella A, Molero R, Leiva-Rebollo R, Pérez-Recuerda R, Borrego JJ. 2021. Identification, resistance to antibiotics and biofilm formation of bacterial strains isolated from a reverse osmosis system of a drinking water treatment plant. Science of The Total Environment 774:145718. doi:10.1016/j.scitotenv.2021.145718

[72] Montaña S, Schramm STJ, Traglia GM, Chiem K, Parmeciano Di Noto G, Almuzara M, Barberis C, Vay C, Quiroga C, Tolmasky ME, et al.. 2016. The genetic analysis of an *Acinetobacter johnsonii* clinical strain evidenced the presence of horizontal genetic transfer. PLoS One 11:e0161528. doi:10.1371/journal.pone.0161528 27548264 PMC4993456

[73] Seifert H, Strate A, Schulze A, Pulverer G. 1993. Vascular catheter-related bloodstream infection due to *Acinetobacter johnsonii* (formerly *Acinetobacter calcoaceticus* var. lwoffi): report of 13 cases. Clin Infect Dis 17:632–636. doi:10.1093/clinids/17.4.632 8268343

[74] Zhang A-N, Gaston JM, Dai CL, Zhao S, Poyet M, Groussin M, Yin X, Li L-G, van Loosdrecht MCM, Topp E, et al.. 2021. An omics-based framework for assessing the health risk of antimicrobial resistance genes. Nat Commun 12:4765. doi:10.1038/s41467-021-25096-3 34362925 PMC8346589

[75] Crocetti GR, Banfield JF, Keller J, Bond PL, Blackall LL. 2002. Glycogen-accumulating organisms in laboratory-scale and full-scale wastewater treatment processesbbThe GenBank accession numbers for the sequences reported in this paper are given in Methods. Microbiology 148:3353–3364. doi:10.1099/00221287-148-11-3353 12427927

[76] McIlroy SJ, Albertsen M, Andresen EK, Saunders AM, Kristiansen R, Stokholm-Bjerregaard M, Nielsen KL, Nielsen PH. 2014. *Candidatus Competibacter*’-lineage genomes retrieved from metagenomes reveal functional metabolic diversity. ISME J 8:613–624. doi:10.1038/ismej.2013.162 24173461 PMC3930307

[77] Ekholm J, Persson F, de Blois M, Modin O, Gustavsson DJI, Pronk M, van Loosdrecht MCM, Wilén B-M. 2024. Microbiome structure and function in parallel full-scale aerobic granular sludge and activated sludge processes. Appl Microbiol Biotechnol 108:334. doi:10.1007/s00253-024-13165-8 38739161 PMC11090927

[78] Narciso-da-Rocha C, Rocha J, Vaz-Moreira I, Lira F, Tamames J, Henriques I, Martinez JL, Manaia CM. 2018. Bacterial lineages putatively associated with the dissemination of antibiotic resistance genes in a full-scale urban wastewater treatment plant. Environ Int 118:179–188. doi:10.1016/j.envint.2018.05.040 29883764

[79] Ong AQ, Philo SE, Taylor A, Hu R, Meschke JS, Fuhrmeister ER. 2024. Targeted sequencing of CTX-M alleles encoding for extended-spectrum beta-lactamases in Seattle Area Wastewater. Environ Sci Technol Lett 11:1017–1024. doi:10.1021/acs.estlett.4c00653

[80] Deshpande AS, Ulahannan N, Pendleton M, Dai X, Ly L, Behr JM, Schwenk S, Liao W, Augello MA, Tyer C, et al.. 2022. Identifying synergistic high-order 3D chromatin conformations from genome-scale nanopore concatemer sequencing. Nat Biotechnol 40:1488–1499. doi:10.1038/s41587-022-01289-z 35637420

[81] Andrews S. 2010. FastQC: a quality control tool for high throughput sequence data. Babraham Bioinformatics.

[82] Bushnell B. 2015. BBMap. sourceforge.net/projects/bbmap.

[83] Nurk S, Meleshko D, Korobeynikov A, Pevzner PA. 2017. metaSPAdes: a new versatile metagenomic assembler. Genome Res 27:824–834. doi:10.1101/gr.213959.116 28298430 PMC5411777

[84] Gurevich A, Saveliev V, Vyahhi N, Tesler G. 2013. QUAST: quality assessment tool for genome assemblies. Bioinformatics 29:1072–1075. doi:10.1093/bioinformatics/btt086 23422339 PMC3624806

[85] Mikheenko A, Prjibelski A, Saveliev V, Antipov D, Gurevich A. 2018. Versatile genome assembly evaluation with QUAST-LG. Bioinformatics 34:i142–i150. doi:10.1093/bioinformatics/bty266 29949969 PMC6022658

[86] Press MO, Wiser AH, Kronenberg ZN, Langford KW, Shakya M, Lo C-C, Mueller KA, Sullivan ST, Chain PSG, Liachko I. 2017. Hi-C deconvolution of a human gut microbiome yields high-quality draft genomes and reveals plasmid-genome interactions. bioRxiv. doi:10.1101/198713:198713

[87] Li H. 2013. Aligning sequence reads, clone sequences and assembly contigs with BWA-MEM. arXiv. https://arxiv.org/abs/1303.3997.

[88] Li H, Handsaker B, Wysoker A, Fennell T, Ruan J, Homer N, Marth G, Abecasis G, Durbin R. 2009. The sequence alignment/map format and SAMtools. Bioinformatics 25:2078–2079. doi:10.1093/bioinformatics/btp352 19505943 PMC2723002

[89] Stewart RD, Auffret MD, Warr A, Wiser AH, Press MO, Langford KW, Liachko I, Snelling TJ, Dewhurst RJ, Walker AW, et al.. 2018. Assembly of 913 microbial genomes from metagenomic sequencing of the cow rumen. Nat Commun 9:870. doi:10.1038/s41467-018-03317-6 29491419 PMC5830445

[90] Aroney STN, Newell RJP, Nissen JN, Camargo AP, Tyson GW, Woodcroft BJ. 2025. CoverM: read alignment statistics for metagenomics. Bioinformatics 41:btaf147. doi:10.1093/bioinformatics/btaf147 40193404 PMC11993303

[91] Chaumeil P-A, Mussig AJ, Hugenholtz P, Parks DH. 2020. GTDB-Tk: a toolkit to classify genomes with the Genome Taxonomy Database. Bioinformatics 36:1925–1927. doi:10.1093/bioinformatics/btz848 PMC7703759 31730192

[92] Camacho C, Coulouris G, Avagyan V, Ma N, Papadopoulos J, Bealer K, Madden TL. 2009. BLAST+: architecture and applications. BMC Bioinformatics 10:421. doi:10.1186/1471-2105-10-421 20003500 PMC2803857

[93] Molano L-A, Hirsch P, Hannig M, Müller R, Keller A. 2025. The PLSDB 2025 update: enhanced annotations and improved functionality for comprehensive plasmid research. Nucleic Acids Res 53:D189–D196. doi:10.1093/nar/gkae1095 39565221 PMC11701622

[94] Wickham H, François R, Henry L, Müller K, Vaughan D. 2023. Dplyr: a grammar of data manipulation. vR package version 1.1.4. https://github.com/tidyverse/dplyr.

[95] Team RC. 2024. R: a language and environment for statistical computing. R Foundation for Statistical Computing, Vienna, Austria. https://www.R-project.org.

[96] Posit team. 2024. RStudio: integrated development environment for R., v2023.12.1+402. Posit Software, PBC, Boston, MA. http://www.posit.co.

[97] Wickham H, Hester J, Bryan J. 2024. Readr: Read Rectangular Text Data. vR package version 2.1.5. https://github.com/tidyverse/readr. https://readr.tidyverse.org.

[98] Wickham H, Averick M, Bryan J, Chang W, McGowan L, François R, Grolemund G, Hayes A, Henry L, Hester J, Kuhn M, Pedersen T, Miller E, Bache S, Müller K, Ooms J, Robinson D, Seidel D, Spinu V, Takahashi K, Vaughan D, Wilke C, Woo K, Yutani H. 2019. Welcome to the Tidyverse. JOSS 4:1686. doi:10.21105/joss.01686

[99] Wickham H. 2016. Ggplot2: elegant graphics for data analysis. Springer, Verlag, New York.

[100] van den Brand T. 2024. Ggh4x: hacks for “ggplot2”. vR package version 0.3.0. https://CRAN.R-project.org/package=ggh4x.

[101] Lin Pedersen T, Crameri F. 2023. Scico: colour palettes based on the scientific colour-maps. vR package version 1.5.0. https://CRAN.R-project.org/package=scico.

[102] Wright K. 2024. Pals: color palettes, colormaps, and tools to evaluate them. vR package version 1.9. https://CRAN.R-project.org/package=pals.

[103] Kassambara A. 2023. Ggpubr: “ggplot2” based publication ready plots. vR package version 0.6.0. https://CRAN.R-project.org/package=ggpubr.

[104] Wilke CO. 2024. Cowplot: streamlined plot theme and plot annotations for “ggplot2”. vR package version 1.1.3. https://CRAN.R-project.org/package=cowplot.

[105] Vu VQ, Friendly M. 2024. Ggbiplot: a grammar of graphics implementation of biplots. vR package version 0.6.2. https://CRAN.R-project.org/package=ggbiplot.

[106] Roberts DW. 2023. Labdsv: ordination and multivarite analysis for ecolog. vR package version 2.1-0. https://CRAN.R-project.org/package=labdsv.

[107] Kassambara A, Mundt F. 2020. Factoextra: extract and visualize the results of multivariate data analyses. vR package version 1.0.7. https://CRAN.R-project.org/package=factoextra.

[108] Oksanen J, Simpson GL, Blanchet FG, Kindt R, Legendre P, Minchin PR, O’Hara RB, Solymos P, Stevens MHH, Szoecs E, et al.. 2022. Vegan: community ecology package. vR package version 2.6-4. https://CRAN.R-project.org/package=vegan.

