# Supplementary material for: Assessing resistome host range across water reclamation in three geographically distinct communities using Hi-C sequencing
Source: Appl Environ Microbiol. 2026 Jul 27;92(8):e00701-26. doi: 10.1128/aem.00701-26 (PMC13488303; doi:10.1128/aem.00701-26)
Supplement: Supplementary file 1 [file aem.00701-26-s0001.pdf]

Assessing Resistome Host Range Across Water Reclamation in Three Geographically Distinct Communities using Hi-C Sequencing

Sarah E. Philo[1], Michael A. Saldana[1], Harmita Golwala[1], Siyi Zhou[2], Lauren B. Stadler[2], Jeseth Delgado Vela[3], Adam L. Smith[1]

[1] Sonny Astani Department of Civil and Environmental Engineering, University of Southern California, Los Angeles, California, USA

[2] Civil and Environmental Engineering, Rice University, Houston, Texas, USA

[3] Department of Civil and Environmental Engineering, Duke University, Durham, North Carolina, USA

Corresponding Author:

Adam L. Smith, PhD, University of Southern California
920 Downey Way, Los Angeles, California, 90089
Tel: +1 213-740-0473
Email: smithada@usc.edu

Supplemental Information

1 Appendix A: Tables and Figures

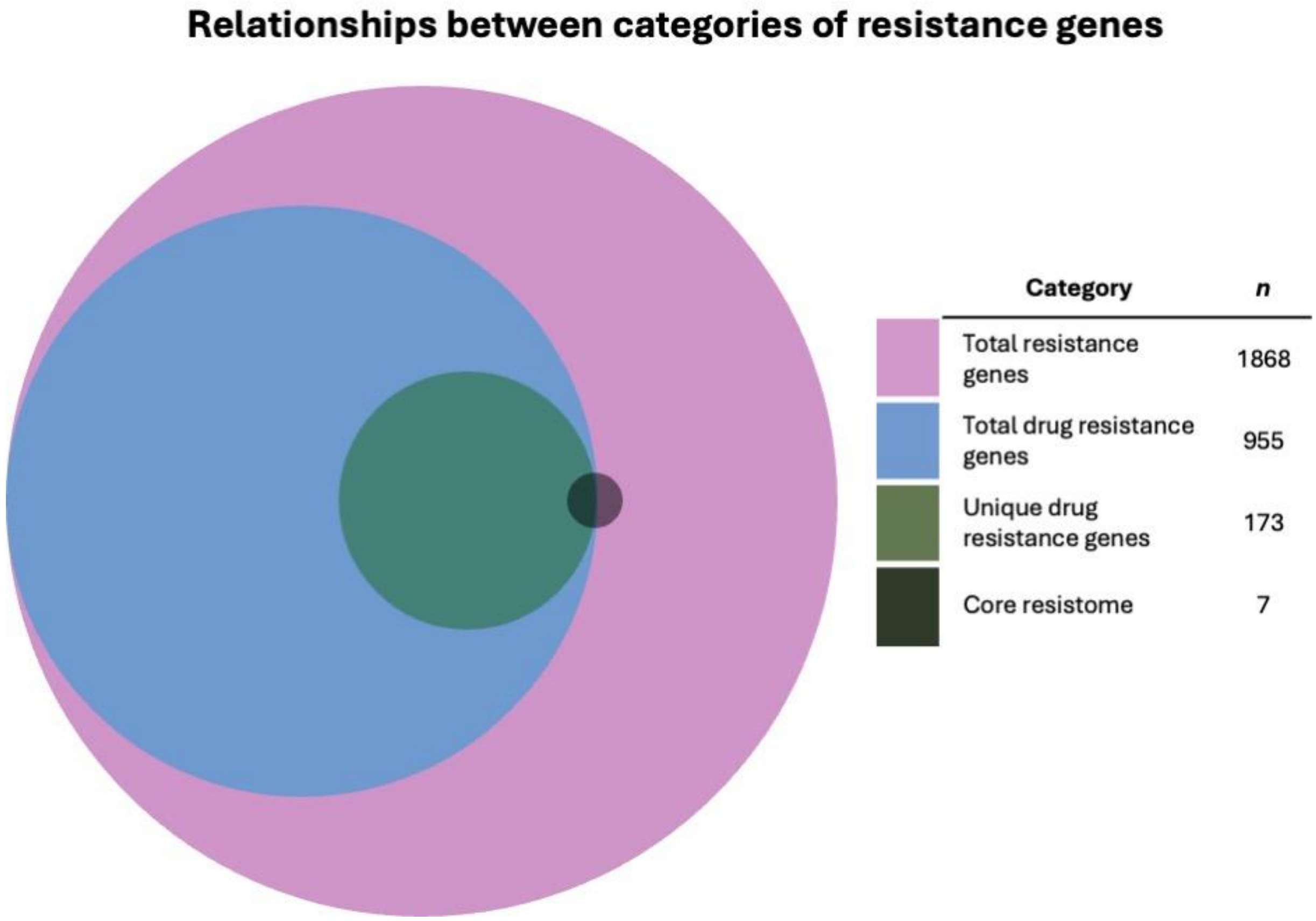

SI Figure 1: Nested Venn Diagram showing the relationships between the different categories of resistance genes analyzed in the current study.

SI Table 1: Sequencing quality information and DNA concentrations for all samples that underwent Hi-C deconvolution. Assembly N50 was obtained with QUAST using the quast.py command and bin coverage of the Hi-C bins was obtained using the genome command of CoverM. DNA concentrations are reported as ng/μL. Concentrations of the Hi-C libraries were obtained using a Qubit 4 Fluorometer with the HS assay and DNA extraction concentrations were obtained using a NanoDrop One (Thermo Fisher Scientific, Waltham, MA, USA). AS = activated sludge.

**North Carolina**						
	Sample	*Assembly N50*	*# bins*	*Bin Coverage*	*DNA Extract Conc.*	*Hi-C Library Conc.*
Influent	1	1199	29	28.56%	42.4	0.165
	2	1139	65	50.46%	149.2	2.62
	3	1274	169	66.95%	32.0	5.86
AS	1	988	68	37.51%	492.8	6.04
	2	956	73	38.01%	3085.8	32.3
	3	1186	47	31.28%	1436.7	30.7
Effluent	1	1636	13	6.66%	25.2	0.444
	2	945	6	6.70%	5.5	0.940
	3	-	-	-	3.6	0.533
**Texas**						
	Sample	*Assembly N50*	*# bins*	*Bin Coverage*	*DNA Extract Conc.*	*Hi-C Library Conc.*
Influent	1	1023	78	52.00%	92.5	44.8
	2	1032	135	62.59%	84.6	22.6
	3	993	56	26.67%	13.2	39.6
AS	1	1049	70	43.86%	438.9	42.0
	2	1032	73	46.62%	732.2	19.4
	3	1138	95	54.18%	1296.3	46.5
Effluent	1	1050	9	7.07%	26.7	0.450
	2	915	81	33.94%	298.7	11.1
	3	993	53	34.49%	13.9	16.3
**California**						
	Sample	*Assembly N50*	*# bins*	*Bin Coverage*	*DNA Extract Conc.*	*Hi-C Library Conc.*
Influent	1	1229	54	46.28%	755.5	3.43
	2	1231	87	45.35%	904.5	3.61
	3	1122	76	43.57%	676.0	3.32
AS	1	1547	141	68.72%	581.1	9.32
	2	1506	142	67.28%	2238.5	3.79
	3	1485	114	73.35%	1154.1	10.3
Effluent	1	-	-	-	152.8	20.6
	2	-	-	-	64.4	13.5
	3	1341	108	40.91%	85.6	28.4

SI Table 2: Antibiotic resistance genes detected in either a bacterial host plasmid or chromosome by Hi-C contact mapping across all samples that underwent the complete deconvolution platform. n_{tot} represents the total number of genes detected in that sample type from that geographic area. Percentages represent the fraction of genes detected in either a microbial plasmid or chromosome from that sample type and geographic location. AS = activated sludge

	North Carolina			Texas			California		
	n_{tot}	Plasmid	Chr.	n_{tot}	Plasmid	Chr.	n_{tot}	Plasmid	Chr.
Influent	223	26<br>11.7%	8<br>3.6%	132	10<br>7.6%	2<br>1.5%	246	24<br>9.8%	10<br>4.1%
AS	56	2<br>3.6%	3<br>5.4%	72	5<br>6.9%	1<br>1.4%	64	6<br>9.4%	9<br>14.1%
Effluent	40	0<br>-	0<br>-	93	0<br>-	4<br>2.10%	29	4<br>13.8%	0<br>-

SI Table 3: Total ARGs and mean relative abundance for each class of antibiotics detected via shotgun sequencing in the samples collected. Individual ARGs were grouped according to the primary class of antibiotic they confer resistance against. Total ARGs is the sum of all individual genes detected in that antibiotic class. Relative abundance was calculated by adjusting the transcripts per million (tpm) assigned reads to that class of antibiotics relative to the total tpm for all assigned AMR reads.

Class	*Total ARGs*	*Mean Relative Abundance*	*95% CI*
Aminoglycoside	112	9.3%	7.9% - 10.8%
Beta-Lactam	320	23.7%	21.5% - 26.0%
Bleomycin	1	1.0%	N/A
Colistin	23	2.5%	1.3% - 3.7%
Efflux	25	1.4%	0.5% - 2.4%
Fosfomycin	9	0.6%	0.2% - 1.1%
Lincosamide	24	1.3%	0.6% - 2.1%
Lincosamide/Streptogramin	5	0.5%	-0.1% - 1.2%
Macrolide	180	27.7%	25.1% - 30.4%
Phenicol	30	2.1%	1.6% - 2.6%
Phenicol/Quinolone	7	0.4%	0.03% - 0.8%
Quinolone	31	4.1%	2.7% - 5.6%
Rifamycin	5	0.8%	0.2% - 1.3%
Sulfonamide	32	22.6%	17.3% - 27.8%
Tetracycline	138	12.1%	10.0% - 14.2%
Trimethoprim	13	3.1%	0.4% - 5.7%

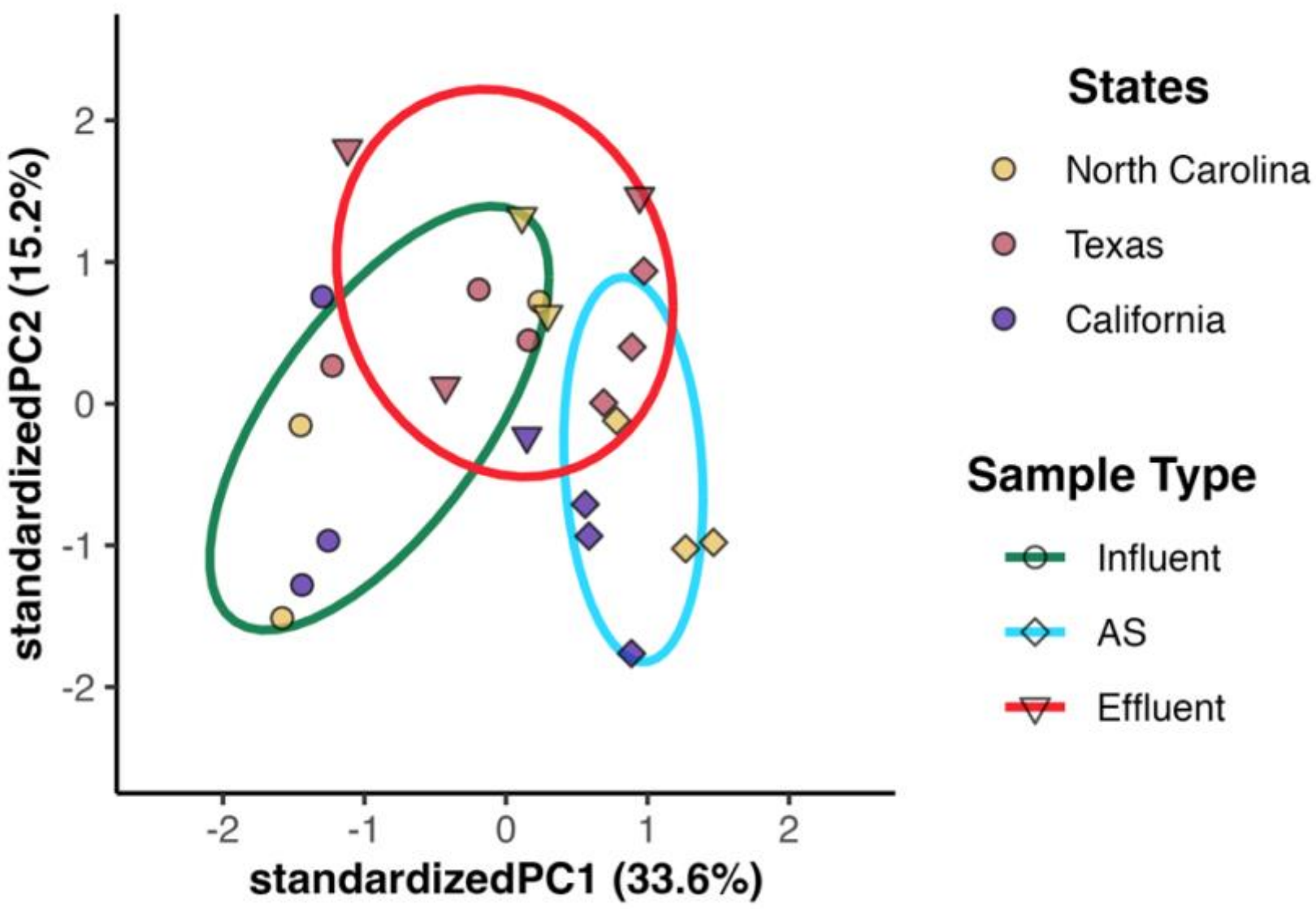

SI Figure 2: Principal component analysis with centering and scaling of the resistome relative abundance at the antibiotic class level. Ellipses represent 68% coverage of the data for each sample type. Prior to PCA, data underwent a Hellinger transformation. PERMANOVA identified significant clustering between sample types ($p < 0.001$), but not between states ($p = 0.11$). AS = activated sludge.

SI Table 4: Core resistome detections and location in bacterial host genomes, either plasmid or chromosome (Chr.), for each sampling location and sample type. "None" were resistance genes annotated on contigs with no informative Hi-C crosslinks or insufficient sequencing depth or quality to identify a bacterial host bin. AS = activated sludge

	North Carolina								
	Influent			*AS*			*Final Effluent*		
	Plasmid	*Chr.*	*None*	*Plasmid*	*Chr.*	*None*	*Plasmid*	*Chr.*	*None*
bla_{OXA}	0	2	17	0	1	4	0	0	3
merE	1	0	8	0	0	7	0	0	4
merP	3	2	13	1	3	8	0	0	3
merR	2	0	15	1	3	4	0	0	4
merT	3	2	17	1	3	7	0	0	5
msr(E)	6	0	1	0	0	3	0	0	2
tet(C)	1	0	2	0	0	3	0	0	2
**Total**	**16**	**6**	**73**	**2**	**10**	**36**	**0**	**0**	**23**
**% of Total**	**16.8**	**6.3**	**76.8**	**4.2**	**20.8**	**75.0**	**0.0**	**0.0**	**100.0**

	Texas								
	Influent			*AS*			*Final Effluent*		
	Plasmid	*Chr.*	*None*	*Plasmid*	*Chr.*	*None*	*Plasmid*	*Chr.*	*None*
bla_{OXA}	0	1	5	0	0	6	0	1	7
merE	3	0	3	0	0	5	0	0	6
merP	4	0	10	0	2	11	0	0	12
merR	1	1	16	0	2	17	0	0	12
merT	4	0	11	0	2	14	0	0	11
msr(E)	1	0	2	0	0	3	0	0	3
tet(C)	1	0	2	0	0	3	0	0	3
**Total**	**14**	**2**	**49**	**0**	**6**	**59**	**0**	**1**	**54**
**% of Total**	**21.5**	**3.1**	**75.4**	**0.0**	**9.2**	**90.8**	**0.0**	**1.8**	**98.2**

	Southern California								
	Influent			*AS*			*Final Effluent*		
	Plasmid	*Chr.*	*None*	*Plasmid*	*Chr.*	*None*	*Plasmid*	*Chr.*	*None*
bla_{OXA}	0	1	17	0	3	10	0	0	3
merE	2	1	8	1	2	4	0	1	4
merP	4	0	9	1	2	5	0	1	3
merR	2	1	8	0	4	8	0	1	3
merT	3	0	10	1	3	7	0	1	2
msr(E)	6	0	0	1	0	2	1	0	0
tet(C)	1	0	2	0	0	3	0	0	1
**Total**	**18**	**3**	**54**	**4**	**14**	**39**	**1**	**4**	**16**
**% of Total**	**24.0**	**4.0**	**72.0**	**7.0**	**24.6**	**68.4**	**4.8**	**19.0**	**76.2**

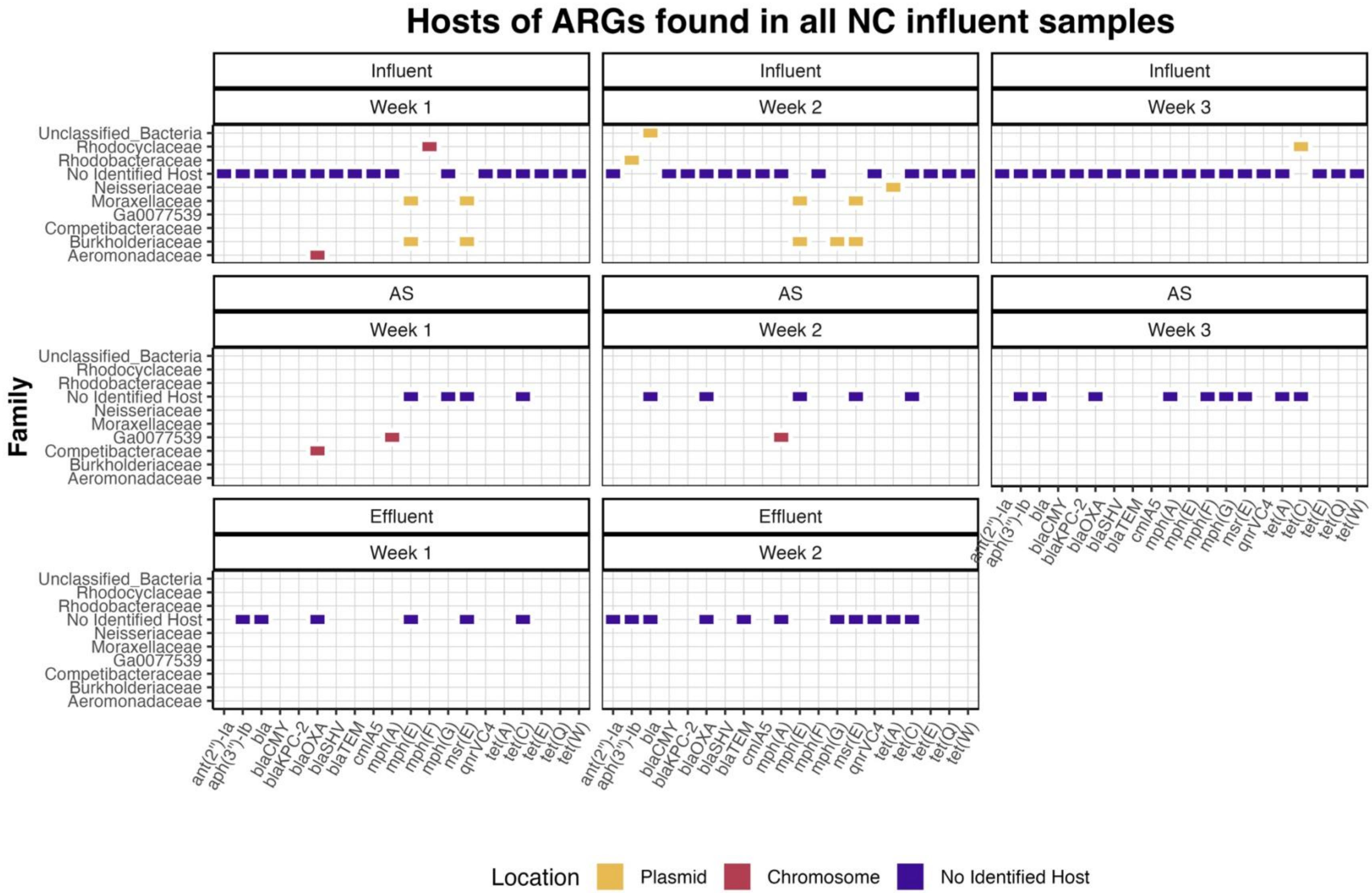

SI Figure 3: The host range of core influent ARGs from North Carolina across treatment. Genes with "No Identified host" were annotated in the assembly but no identified associations with bacterial chromosomes or plasmids. Unclassified hosts were identified as plasmids and/or bacterial chromosomes crosslinked with resistance genes in the assemblies that did not meet sequencing quality criteria during classification. AS = activated sludge

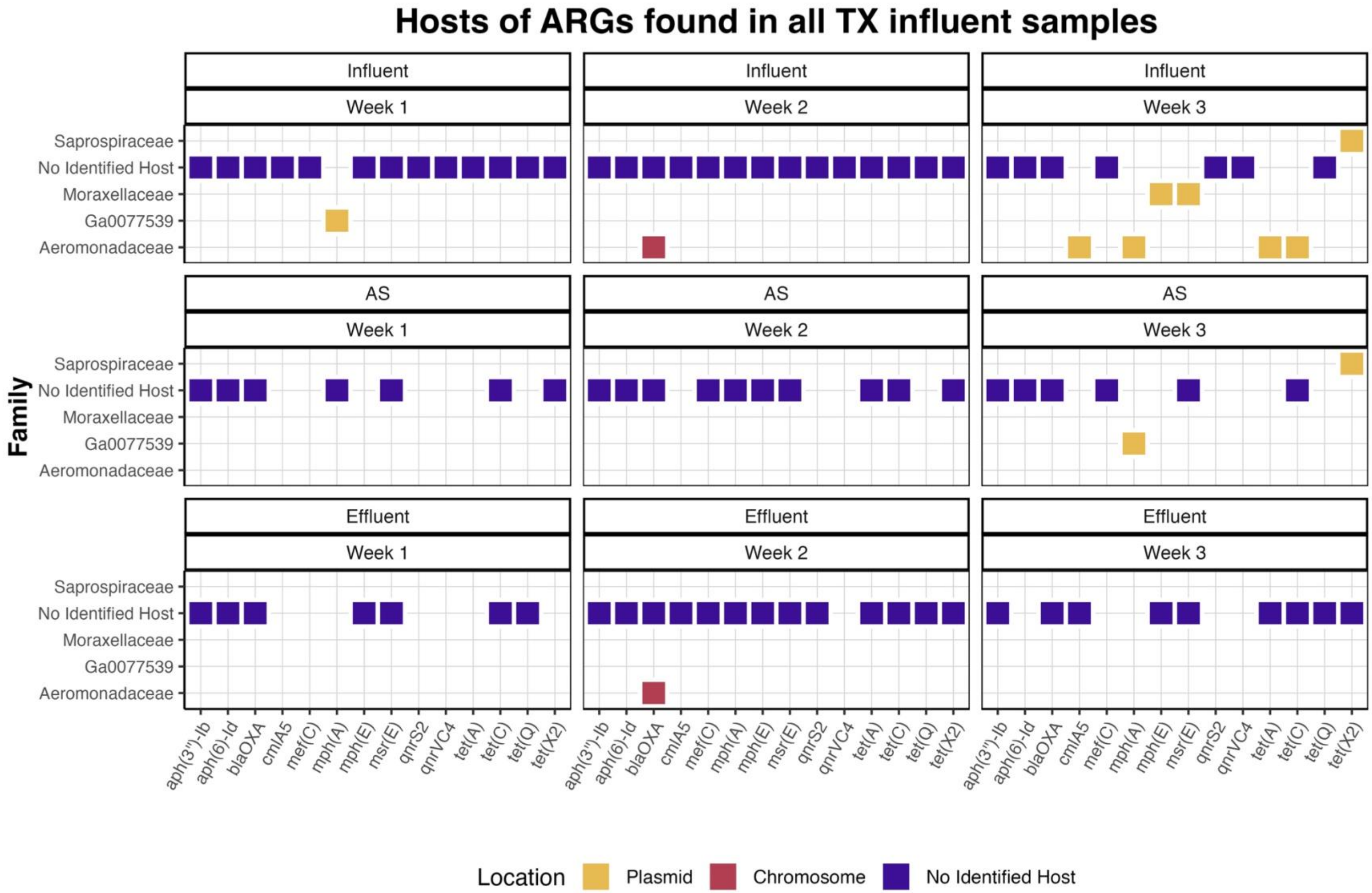

SI Figure 4: The host range of core influent ARGs from Texas across wastewater treatment. Genes with "No Identified host" were annotated in the assembly but no identified associations with bacterial chromosomes or plasmids. Unclassified hosts were identified as plasmids and/or bacterial chromosomes crosslinked with resistance genes in the assemblies that did not meet sequencing quality criteria during classification. AS = activated sludge.

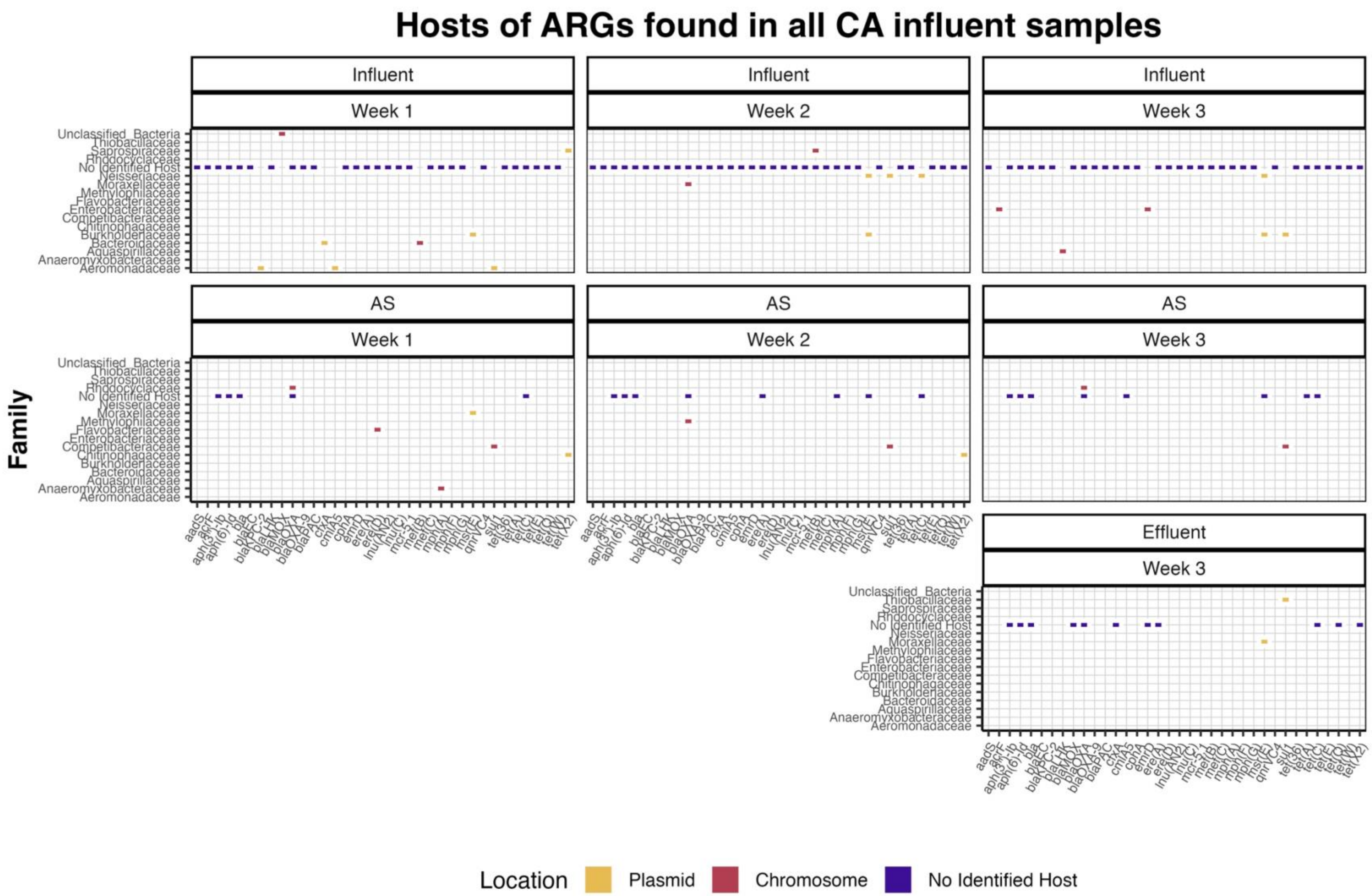

SI Figure 5: The host range of core influent ARGs from California across wastewater treatment. Genes with "No Identified host" were annotated in the assembly but no identified associations with bacterial chromosomes or plasmids. Unclassified hosts were identified as plasmids and/or bacterial chromosomes crosslinked with resistance genes in the assemblies that did not meet sequencing quality criteria during classification. AS = activated sludge.

SI Table 5: Location in bacterial genome and stage of treatment for the four families representing more than half of all bacterial hosts identified by Hi-C contact maps. Chrom. indicates the hosted resistance gene is in the bacterial chromosome, and Plas. indicates the hosted resistance gene is in a plasmid. AS = activated sludge.

	Influent		AS		Effluent	
Family	*Chrom.*	*Plas.*	*Chrom.*	*Plas.*	*Chrom.*	*Plas.*
Aeromonadaceae	4	8	-	-	1	-
Burkholderiaceae	-	17	-	1	-	-
Moraxellaceae	5	10	1	3	-	3
Neisseriaceae	-	8	-	-	-	-

SI Table 6: Dates and volumes of samples collected from the three water reclamation facilities included in the study.

	North Carolina		Texas		California	
	Date	*Volume*	*Date*	*Volume*	*Date*	*Volume*
Influent	30-Apr-24	2.0 mL x 3	30-Apr-24	2.0 mL x 3	28-Apr-24	2.0 mL x 3
	14-May-24		14-May-24		12-May-24	
	23-May-24		21-May-24		19-May-24	
Activated Sludge	30-Apr-24	2.0 mL x 3	30-Apr-24	2.0 mL x 3	29-Apr-24	2.0 mL x 3
	14-May-24		14-May-24		13-May-24	
	23-May-24		21-May-24		20-May-24	
Final Effluent	30-Apr-24	500 mL x 3	30-Apr-24	500 mL x 3	28-Apr-24	500 mL x 3
	14-May-24		14-May-24		12-May-24	
	23-May-24		21-May-24		19-May-24	

2 Appendix B: Supplemental Methods

2.1 Genome assembly commands

FastQC

```
infile=$(ls *_001.fastq.gz | sort | sed -n "${SLURM_ARRAY_TASK_ID}p")
base=$(basename "${infile}" _001.fastq.gz)

# Fastqc of the reads
fastqc -t 32 "${infile}"
```

metaSPADES

```
infile_R1=$(ls *_R1_trim.fq | sort | sed -n "${SLURM_ARRAY_TASK_ID}p")
infile_R2=$(echo "$infile_R1" | sed 's/_R1_/_R2_/')
base=$(basename "${infile_R1}" _R1_trim.fq)

# Run MetaSPAdes for metagenomic assembly
spades.py --meta --threads 16 --memory 1390 \
   -1 "${infile_R1}" \
   -2 "${infile_R2}" \
   -o "${output_directory}/${base}_spades_output"
```

BBDUK

```
infile_R1=$(ls *_R1_001.fastq.gz | sort | sed -n "${SLURM_ARRAY_TASK_ID}p")
infile_R2=$(echo "$infile_R1" | sed 's/_R1_/_R2_/')
base=$(basename "${infile_R1}" _R1_001.fastq.gz)

# bbduk adapter removal for paired-end reads
bbduk.sh in1="$infile_R1" in2="$infile_R2" out1="${base}_R1_trim.fq"
out2="${base}_R2_trim.fq" \
ref=$PATH/bbmap/resources/adapters.fa \
threads=32 ktrim=r k=24 mink=11 hdist=1 tpe tbo qtrim=rl trimq=10 minlength=50
chastityfilter=True
```

3 SI Data

3.1 All genes with informative species level host information

Gene ID	Order	Family	Genus	Species	State	Sample	Week
blaOXA	Enterobacterales	Aeromonadaceae	Aeromonas	Aeromonas caviae	NC	Influent	Week 1
blaOXA	Enterobacterales	Aeromonadaceae	Aeromonas	Aeromonas caviae	NC	Influent	Week 1
icr-Mo	Pseudomonadales	Moraxellaceae	Moraxella_A	Moraxella_A sp002478835	NC	Influent	Week 1
mph(E)	Pseudomonadales	Moraxellaceae	Acinetobacter	Acinetobacter johnsonii	NC	Influent	Week 1
mph(E)	Burkholderiales	Burkholderiaceae	Comamonas	Comamonas denitrificans_A	NC	Influent	Week 1
mph(F)	Burkholderiales	Rhodocyclaceae	Azonexus	Unclassified Azonexus	NC	Influent	Week 1
msr(E)	Pseudomonadales	Moraxellaceae	Acinetobacter	Acinetobacter johnsonii	NC	Influent	Week 1
msr(E)	Burkholderiales	Burkholderiaceae	Comamonas	Comamonas denitrificans_A	NC	Influent	Week 1
merP	Burkholderiales	Rhodocyclaceae	Azonexus	Unclassified Azonexus	NC	Influent	Week 1
merT	Burkholderiales	Rhodocyclaceae	Azonexus	Unclassified Azonexus	NC	Influent	Week 1
tet(39)	Pseudomonadales	Moraxellaceae	Acinetobacter	Acinetobacter johnsonii	NC	Influent	Week 1
aph(3'')-Ib	Rhodobacterales	Rhodobacteraceae	Paracoccus	Unclassified Paracoccus	NC	Influent	Week 2
aph(6)-Id	Rhodobacterales	Rhodobacteraceae	Paracoccus	Unclassified Paracoccus	NC	Influent	Week 2
bla	Unclassified Bacteria	Unclassified Bacteria	Unclassified Bacteria	Unclassified Bacteria	NC	Influent	Week 2
blaLHK	Burkholderiales	Aquaspirillaceae	Laribacter	Laribacter hongkongensis	NC	Influent	Week 2
icr-Mo	Pseudomonadales	Moraxellaceae	Moraxella_A	Moraxella_A sp002478835	NC	Influent	Week 2
hdeD-GI	Burkholderiales	Burkholderiaceae	Ottowia	Ottowia sp018060485	NC	Influent	Week 2
shsP	Burkholderiales	Burkholderiaceae	Ottowia	Ottowia sp018060485	NC	Influent	Week 2
trxLHR	Burkholderiales	Burkholderiaceae	Ottowia	Ottowia sp018060485	NC	Influent	Week 2
yfdX1	Burkholderiales	Burkholderiaceae	Ottowia	Ottowia sp018060485	NC	Influent	Week 2
yfdX2	Burkholderiales	Burkholderiaceae	Ottowia	Ottowia sp018060485	NC	Influent	Week 2
lnu(B)	Fusobacteriales	Leptotrichiaceae	JAGOYM01	JAGOYM01 sp018059165	NC	Influent	Week 2
lsa(E)	Fusobacteriales	Leptotrichiaceae	JAGOYM01	JAGOYM01 sp018059165	NC	Influent	Week 2

ere(A)	Burkholderiales	Burkholderiaceae	Brachymonas	Brachymonas sp018054915	NC	Influent	Week 2
mef(C)	Burkholderiales	Burkholderiaceae	Brachymonas	Brachymonas sp018054915	NC	Influent	Week 2
mph(E)	Burkholderiales	Burkholderiaceae	Brachymonas	Brachymonas sp018054915	NC	Influent	Week 2
mph(E)	Burkholderiales	Burkholderiaceae	Brachymonas	Brachymonas sp017993135	NC	Influent	Week 2
mph(E)	Pseudomonadales	Moraxellaceae	Acinetobacter	Acinetobacter johnsonii	NC	Influent	Week 2
mph(E)	Pseudomonadales	Moraxellaceae	Moraxella_A	Moraxella_A sp002478835	NC	Influent	Week 2
mph(G)	Burkholderiales	Burkholderiaceae	Brachymonas	Brachymonas sp018054915	NC	Influent	Week 2
msr(E)	Burkholderiales	Burkholderiaceae	Brachymonas	Brachymonas sp018054915	NC	Influent	Week 2
msr(E)	Burkholderiales	Burkholderiaceae	Brachymonas	Brachymonas sp017993135	NC	Influent	Week 2
msr(E)	Pseudomonadales	Moraxellaceae	Acinetobacter	Acinetobacter johnsonii	NC	Influent	Week 2
msr(E)	Pseudomonadales	Moraxellaceae	Moraxella_A	Moraxella_A sp002478835	NC	Influent	Week 2
merD	Burkholderiales	Burkholderiaceae	Ottowia	Ottowia sp018060485	NC	Influent	Week 2
merE	Burkholderiales	Burkholderiaceae	Ottowia	Ottowia sp018060485	NC	Influent	Week 2
merF	Burkholderiales	Burkholderiaceae	Comamonas	Comamonas denitrificans_A	NC	Influent	Week 2
merP	Pseudomonadales	Pseudomonadaceae	Pseudomonas_E	Pseudomonas_E veronii	NC	Influent	Week 2
merP	Burkholderiales	Burkholderiaceae	Comamonas	Comamonas denitrificans_A	NC	Influent	Week 2
merP	Burkholderiales	Burkholderiaceae	Ottowia	Ottowia sp018060485	NC	Influent	Week 2
merR	Burkholderiales	Burkholderiaceae	Ottowia	Ottowia sp018060485	NC	Influent	Week 2
merT	Pseudomonadales	Pseudomonadaceae	Pseudomonas_E	Pseudomonas_E veronii	NC	Influent	Week 2
merT	Burkholderiales	Burkholderiaceae	Comamonas	Comamonas denitrificans_A	NC	Influent	Week 2
merT	Burkholderiales	Burkholderiaceae	Ottowia	Ottowia sp018060485	NC	Influent	Week 2
cmx	Xanthomonadales	Xanthomonadaceae	Lysobacter_B	Lysobacter_B sp014879375	NC	Influent	Week 2
floR	Rhodobacterales	Rhodobacteraceae	Paracoccus	Unclassified Paracoccus	NC	Influent	Week 2
tet(39)	Pseudomonadales	Moraxellaceae	Acinetobacter	Acinetobacter johnsonii	NC	Influent	Week 2
tet(A)	Burkholderiales	Neisseriaceae	Neisseria	Neisseria suis	NC	Influent	Week 2
tet(G)	Rhodobacterales	Rhodobacteraceae	Paracoccus	Unclassified Paracoccus	NC	Influent	Week 2
merC	Nitrospirales	Nitrospiraceae	Nitrospira_A	Nitrospira_A defluvii_A	NC	Influent	Week 3
merP	Nitrospirales	Nitrospiraceae	Nitrospira_A	Nitrospira_A defluvii_A	NC	Influent	Week 3
merR	Nitrospirales	Nitrospiraceae	Nitrospira_A	Nitrospira_A defluvii_A	NC	Influent	Week 3

merT	Nitrospirales	Nitrospiraceae	Nitrospira_A	Nitrospira_A defluvii_A	NC	Influent	Week 3
sul1	Nitrospirales	Nitrospiraceae	Nitrospira_A	Nitrospira_A defluvii_A	NC	Influent	Week 3
tet(C)	Burkholderiales	Rhodocyclaceae	Fen-999	Fen-999 sp016704145	NC	Influent	Week 3
blaOXA	Competibacterales	Competibacteraceae	Competibacter_A	Unclassified Competibacter_A	NC	AS	Week 1
mph(A)	Polyangiales	Ga0077539	CAISKJ01	Unclassified CAISKJ01	NC	AS	Week 1
sul1	Nitrospirales	Nitrospiraceae	Nitrospira_A	Nitrospira_A defluvii_A	NC	AS	Week 1
mph(A)	Polyangiales	Ga0077539	CAISKJ01	Unclassified CAISKJ01	NC	AS	Week 2
merC	Nitrospirales	Nitrospiraceae	Nitrospira_A	Nitrospira_A defluvii_A	NC	AS	Week 2
merP	Nitrospirales	Nitrospiraceae	Nitrospira_A	Nitrospira_A defluvii_A	NC	AS	Week 2
merR	Nitrospirales	Nitrospiraceae	Nitrospira_A	Nitrospira_A defluvii_A	NC	AS	Week 2
merT	Nitrospirales	Nitrospiraceae	Nitrospira_A	Nitrospira_A defluvii_A	NC	AS	Week 2
sul1	Nitrospirales	Nitrospiraceae	Nitrospira_A	Nitrospira_A defluvii_A	NC	AS	Week 2
merC	Burkholderiales	Rhodocyclaceae	Accumulibacter	Unclassified Accumulibacter	NC	AS	Week 3
merP	Unclassified Bacteria	Unclassified Bacteria	Unclassified Bacteria	Unclassified Bacteria	NC	AS	Week 3
merP	Burkholderiales	Burkholderiaceae	JADKHE01	Unclassified JADKHE01	NC	AS	Week 3
merP	Burkholderiales	Rhodocyclaceae	Accumulibacter	Unclassified Accumulibacter	NC	AS	Week 3
merR	Unclassified Bacteria	Unclassified Bacteria	Unclassified Bacteria	Unclassified Bacteria	NC	AS	Week 3
merR	Burkholderiales	Burkholderiaceae	JADKHE01	Unclassified JADKHE01	NC	AS	Week 3
merR	Burkholderiales	Rhodocyclaceae	Accumulibacter	Unclassified Accumulibacter	NC	AS	Week 3
merT	Unclassified Bacteria	Unclassified Bacteria	Unclassified Bacteria	Unclassified Bacteria	NC	AS	Week 3
merT	Burkholderiales	Burkholderiaceae	JADKHE01	Unclassified JADKHE01	NC	AS	Week 3
merT	Burkholderiales	Rhodocyclaceae	Accumulibacter	Unclassified Accumulibacter	NC	AS	Week 3
mph(A)	Polyangiales	Ga0077539	CAISKJ01	Unclassified CAISKJ01	TX	Influent	Week 1
merA	Burkholderiales	Usitatibacteraceae	7-Feb	Unclassified FEB-7	TX	Influent	Week 1
merE	Burkholderiales	Usitatibacteraceae	7-Feb	Unclassified FEB-7	TX	Influent	Week 1
merP	Burkholderiales	Usitatibacteraceae	7-Feb	Unclassified FEB-7	TX	Influent	Week 1
merR	Burkholderiales	Burkholderiaceae	Ottowia	Ottowia sp001897615	TX	Influent	Week 1
merT	Burkholderiales	Usitatibacteraceae	7-Feb	Unclassified FEB-7	TX	Influent	Week 1

sul1	Palsa-1104	JAEUJZ01	JAEUKA01	JAEUKA01 sp937868485	TX	Influent	Week 1
blaOXA	Enterobacterales	Aeromonadaceae	Aeromonas	Aeromonas caviae	TX	Influent	Week 2
merD	Burkholderiales	Burkholderiaceae	Unclassified Burkholderiaceae	Unclassified Burkholderiaceae	TX	Influent	Week 2
merE	Burkholderiales	Burkholderiaceae	Unclassified Burkholderiaceae	Unclassified Burkholderiaceae	TX	Influent	Week 2
merF	Burkholderiales	Burkholderiaceae	Unclassified Burkholderiaceae	Unclassified Burkholderiaceae	TX	Influent	Week 2
merP	Burkholderiales	Usitatibacteraceae	7-Feb	Unclassified FEB-7	TX	Influent	Week 2
merP	Burkholderiales	Burkholderiaceae	Unclassified Burkholderiaceae	Unclassified Burkholderiaceae	TX	Influent	Week 2
merT	Burkholderiales	Usitatibacteraceae	7-Feb	Unclassified FEB-7	TX	Influent	Week 2
merT	Burkholderiales	Burkholderiaceae	Unclassified Burkholderiaceae	Unclassified Burkholderiaceae	TX	Influent	Week 2
sul1	Palsa-1104	JAEUJZ01	JAEUKA01	JAEUKA01 sp937868485	TX	Influent	Week 2
blaMOX	Enterobacterales	Aeromonadaceae	Aeromonas	Aeromonas caviae	TX	Influent	Week 3
yfdX2	Burkholderiales	Burkholderiaceae	Acidovorax	Acidovorax temperans	TX	Influent	Week 3
mph(A)	Enterobacterales	Aeromonadaceae	Aeromonas	Aeromonas caviae	TX	Influent	Week 3
mph(E)	Pseudomonadales	Moraxellaceae	Moraxella_A	Moraxella_A sp002478835	TX	Influent	Week 3
msr(E)	Pseudomonadales	Moraxellaceae	Moraxella_A	Moraxella_A sp002478835	TX	Influent	Week 3
merD	Burkholderiales	Burkholderiaceae	Acidovorax	Acidovorax temperans	TX	Influent	Week 3
merE	Burkholderiales	Burkholderiaceae	Acidovorax	Acidovorax temperans	TX	Influent	Week 3
merF	Burkholderiales	Burkholderiaceae	Acidovorax	Acidovorax temperans	TX	Influent	Week 3
merP	Burkholderiales	Burkholderiaceae	Acidovorax	Acidovorax temperans	TX	Influent	Week 3
merR	Burkholderiales	Burkholderiaceae	Acidovorax	Acidovorax temperans	TX	Influent	Week 3
merT	Burkholderiales	Burkholderiaceae	Acidovorax	Acidovorax temperans	TX	Influent	Week 3
cmlA5	Enterobacterales	Aeromonadaceae	Tolumonas	Tolumonas sp008015085	TX	Influent	Week 3
tet(A)	Enterobacterales	Aeromonadaceae	Tolumonas	Tolumonas sp008015085	TX	Influent	Week 3
tet(C)	Enterobacterales	Aeromonadaceae	Tolumonas	Tolumonas sp008015085	TX	Influent	Week 3
tet(X2)	Chitinophagales	Saprospiraceae	Parvibacillus	Unclassified Parvibacillus	TX	Influent	Week 3
blaVEB-9	2-12-FULL-35-15	2-12-FULL-35-15	2-12-FULL-35-15	Unclassified 2-12-FULL-35-15	TX	AS	Week 1

merP	Competibacterales	Competibacteraceae	Competibacter_A	Unclassified Competibacter_A	TX	AS	Week 1
merR	Competibacterales	Competibacteraceae	Competibacter_A	Unclassified Competibacter_A	TX	AS	Week 1
merT	Competibacterales	Competibacteraceae	Competibacter_A	Unclassified Competibacter_A	TX	AS	Week 1
sul1	Palsa-1104	JAEUJZ01	JAEUKA01	JAEUKA01 sp937868485	TX	AS	Week 1
merP	Competibacterales	Competibacteraceae	RXIW01	RXIW01 sp003989065	TX	AS	Week 2
merR	Competibacterales	Competibacteraceae	RXIW01	RXIW01 sp003989065	TX	AS	Week 2
merT	Competibacterales	Competibacteraceae	RXIW01	RXIW01 sp003989065	TX	AS	Week 2
sul1	Palsa-1104	JAEUJZ01	JAEUKA01	JAEUKA01 sp937868485	TX	AS	Week 2
mph(A)	Polyangiales	Ga0077539	CAISKJ01	Unclassified CAISKJ01	TX	AS	Week 3
sul1	Palsa-1104	JAEUJZ01	JAEUKA01	JAEUKA01 sp937868485	TX	AS	Week 3
tet(X2)	Chitinophagales	Saprospiraceae	Parvibacillus	Unclassified Parvibacillus	TX	AS	Week 3
blaOXA	Enterobacterales	Aeromonadaceae	Aeromonas	Aeromonas caviae	TX	Effluent	Week 2
cblA	Unclassified Bacteria	Unclassified Bacteria	Unclassified Bacteria	Unclassified Bacteria	TX	Effluent	Week 2
lnu(AN2)	Bacteroidales	Bacteroidaceae	Bacteroides_I	Bacteroides_I graminisolvens	TX	Effluent	Week 2
mef(En2)	Bacteroidales	Bacteroidaceae	Bacteroides_I	Bacteroides_I graminisolvens	TX	Effluent	Week 2
aadA10	Burkholderiales	Rhodocyclaceae	Propionivibrio	Unclassified Propionivibrio	CA	Influent	Week 1
blaGES-5	Enterobacterales	Aeromonadaceae	Tolumonas	Tolumonas sp008015085	CA	Influent	Week 1
blaKPC-2	Enterobacterales	Aeromonadaceae	Tolumonas	Tolumonas sp008015085	CA	Influent	Week 1
blaMOX	Unclassified Bacteria	Unclassified Bacteria	Unclassified Bacteria	Unclassified Bacteria	CA	Influent	Week 1
cfxA	Bacteroidales	Bacteroidaceae	Bacteroides_I	Bacteroides_I graminisolvens	CA	Influent	Week 1
hdeD-GI	Burkholderiales	Burkholderiaceae	Ottowia	Ottowia beijingensis	CA	Influent	Week 1
mef(B)	Bacteroidales	Bacteroidaceae	Bacteroides_I	Bacteroides_I graminisolvens	CA	Influent	Week 1
msr(E)	Burkholderiales	Burkholderiaceae	Brachymonas	Brachymonas sp018054915	CA	Influent	Week 1
merD	Burkholderiales	Rhodocyclaceae	Azonexus	Azonexus sp016790885	CA	Influent	Week 1
merD	Pseudomonadales	Pseudomonadaceae	Pseudomonas_E	Unclassified Pseudomonas_E	CA	Influent	Week 1

merE	Pseudomonadales	Pseudomonadaceae	Pseudomonas_E	Unclassified Pseudomonas_E	CA	Influent	Week 1
merF	Pseudomonadales	Pseudomonadaceae	Pseudomonas_E	Unclassified Pseudomonas_E	CA	Influent	Week 1
merP	Pseudomonadales	Pseudomonadaceae	Pseudomonas_E	Unclassified Pseudomonas_E	CA	Influent	Week 1
merR	Burkholderiales	Rhodocyclaceae	Propionivibrio	Unclassified Propionivibrio	CA	Influent	Week 1
merR	Pseudomonadales	Pseudomonadaceae	Pseudomonas_E	Unclassified Pseudomonas_E	CA	Influent	Week 1
merT	Pseudomonadales	Pseudomonadaceae	Pseudomonas_E	Unclassified Pseudomonas_E	CA	Influent	Week 1
cmlA5	Enterobacterales	Aeromonadaceae	Tolumonas	Tolumonas sp008015085	CA	Influent	Week 1
sul1	Enterobacterales	Aeromonadaceae	Tolumonas	Tolumonas sp008015085	CA	Influent	Week 1
tet(X2)	Chitinophagales	Saprospiraceae	Parvibacillus	Parvibacillus calidus	CA	Influent	Week 1
dfrA1	Burkholderiales	Rhodocyclaceae	Propionivibrio	Unclassified Propionivibrio	CA	Influent	Week 1
blaOXA	Pseudomonadales	Moraxellaceae	Acinetobacter	Acinetobacter sp002296655	CA	Influent	Week 2
icr-Mo	Unclassified Bacteria	Unclassified Bacteria	Unclassified Bacteria	Unclassified Bacteria	CA	Influent	Week 2
mef(B)	Chitinophagales	Saprospiraceae	Parvibacillus	Parvibacillus calidus	CA	Influent	Week 2
mph(E)	Burkholderiales	Burkholderiaceae	Brachymonas	Brachymonas sp017996515	CA	Influent	Week 2
mph(E)	Burkholderiales	Burkholderiaceae	Brachymonas	Brachymonas sp018054915	CA	Influent	Week 2
mph(E)	Burkholderiales	Neisseriaceae	Neisseria	Neisseria sp937919795	CA	Influent	Week 2
msr(E)	Burkholderiales	Burkholderiaceae	Brachymonas	Brachymonas sp017996515	CA	Influent	Week 2
msr(E)	Burkholderiales	Burkholderiaceae	Brachymonas	Brachymonas sp018054915	CA	Influent	Week 2
msr(E)	Burkholderiales	Neisseriaceae	Neisseria	Neisseria sp937919795	CA	Influent	Week 2
merF	Pseudomonadales	Pseudomonadaceae	Pseudomonas_E	Unclassified Pseudomonas_E	CA	Influent	Week 2
merP	Pseudomonadales	Pseudomonadaceae	Pseudomonas_E	Unclassified Pseudomonas_E	CA	Influent	Week 2
merP	Pseudomonadales	Pseudomonadaceae	Pseudomonas_E	Unclassified Pseudomonas_E	CA	Influent	Week 2
merR	Pseudomonadales	Pseudomonadaceae	Pseudomonas_E	Unclassified Pseudomonas_E	CA	Influent	Week 2
merT	Pseudomonadales	Pseudomonadaceae	Pseudomonas_E	Unclassified Pseudomonas_E	CA	Influent	Week 2

qacEdelta1	Burkholderiales	Neisseriaceae	Neisseria	Neisseria sp937919795	CA	Influent	Week 2
sul1	Burkholderiales	Neisseriaceae	Neisseria	Neisseria sp937919795	CA	Influent	Week 2
tet(C)	Burkholderiales	Neisseriaceae	Neisseria	Neisseria sp937919795	CA	Influent	Week 2
arsR	Enterobacterales	Enterobacteriaceae	Escherichia	Unclassified Escherichia	CA	Influent	Week 3
blaBRO-2	Pseudomonadales	Moraxellaceae	Moraxella	Unclassified Moraxella	CA	Influent	Week 3
blaLHK	Burkholderiales	Aquaspirillaceae	Laribacter	Laribacter hongkongensis	CA	Influent	Week 3
icr-Mo	Pseudomonadales	Moraxellaceae	Moraxella_A	Moraxella_A sp002478835	CA	Influent	Week 3
acrF	Enterobacterales	Enterobacteriaceae	Escherichia	Unclassified Escherichia	CA	Influent	Week 3
emrD	Enterobacterales	Enterobacteriaceae	Escherichia	Unclassified Escherichia	CA	Influent	Week 3
mph(E)	Burkholderiales	Burkholderiaceae	Brachymonas	Brachymonas sp017996515	CA	Influent	Week 3
mph(E)	Burkholderiales	Neisseriaceae	Neisseria	Unclassified Neisseria	CA	Influent	Week 3
msr(E)	Burkholderiales	Burkholderiaceae	Brachymonas	Brachymonas sp017996515	CA	Influent	Week 3
msr(E)	Burkholderiales	Neisseriaceae	Neisseria	Unclassified Neisseria	CA	Influent	Week 3
merE	Pseudomonadales	Pseudomonadaceae	Pseudomonas_E	Unclassified Pseudomonas_E	CA	Influent	Week 3
merE	Burkholderiales	Rhodocyclaceae	Propionivibrio	Unclassified Propionivibrio	CA	Influent	Week 3
merF	Pseudomonadales	Pseudomonadaceae	Pseudomonas_E	Unclassified Pseudomonas_E	CA	Influent	Week 3
merP	Pseudomonadales	Pseudomonadaceae	Pseudomonas_E	Unclassified Pseudomonas_E	CA	Influent	Week 3
merT	Pseudomonadales	Pseudomonadaceae	Pseudomonas_E	Unclassified Pseudomonas_E	CA	Influent	Week 3
fieF	Enterobacterales	Enterobacteriaceae	Escherichia	Unclassified Escherichia	CA	Influent	Week 3
qacEdelta1	Burkholderiales	Burkholderiaceae	Brachymonas	Brachymonas sp018054915	CA	Influent	Week 3
sul1	Burkholderiales	Burkholderiaceae	Brachymonas	Brachymonas sp018054915	CA	Influent	Week 3
sul2	Burkholderiales	Neisseriaceae	Neisseria	Unclassified Neisseria	CA	Influent	Week 3
tet(O)	Peptostreptococcales	Filifactoraceae	Acetoanaerobium	Acetoanaerobium noterae	CA	Influent	Week 3
blaOXA	Burkholderiales	Rhodocyclaceae	Rugosibacter	Rugosibacter sp002422995	CA	AS	Week 1
mcr-5	Pseudomonadales	Moraxellaceae	UBA2031	Unclassified UBA2031	CA	AS	Week 1
ere(D)	Flavobacteriales	Flavobacteriaceae	Flavobacterium	Unclassified Flavobacterium	CA	AS	Week 1
mph(A)	Myxococcales	Anaeromyxobacteraceae	JADKCZ01	Unclassified JADKCZ01	CA	AS	Week 1

msr(E)	Pseudomonadales	Moraxellaceae	UBA2031	Unclassified UBA2031	CA	AS	Week 1
merC	Burkholderiales	Burkholderiaceae	Ottowia	Unclassified Ottowia	CA	AS	Week 1
merD	Burkholderiales	Burkholderiaceae	Ottowia	Unclassified Ottowia	CA	AS	Week 1
merE	Burkholderiales	Burkholderiaceae	Ottowia	Unclassified Ottowia	CA	AS	Week 1
merP	Burkholderiales	Burkholderiaceae	Ottowia	Unclassified Ottowia	CA	AS	Week 1
merR	Burkholderiales	Burkholderiaceae	Ottowia	Unclassified Ottowia	CA	AS	Week 1
merT	Burkholderiales	Burkholderiaceae	Ottowia	Unclassified Ottowia	CA	AS	Week 1
sul1	Competibacterales	Competibacteraceae	Competibacter	Competibacter phosphatis	CA	AS	Week 1
tet(X2)	Chitinophagales	Chitinophagaceae	Edaphocola	Unclassified Edaphocola	CA	AS	Week 1
blaOXA	Burkholderiales	Methylophilaceae	Methylotenera	Methylotenera sp029549705	CA	AS	Week 2
blaPAU	Burkholderiales	Burkholderiaceae	Aquabacterium_A	Aquabacterium_A sp017999135	CA	AS	Week 2
chrA	Burkholderiales	Burkholderiaceae	JADKHE01	Unclassified JADKHE01	CA	AS	Week 2
mcr-5	Pseudomonadales	Moraxellaceae	UBA2031	Unclassified UBA2031	CA	AS	Week 2
merA	Burkholderiales	Rhodocyclaceae	Methyloversatilis	Unclassified Methyloversatilis	CA	AS	Week 2
merC	Burkholderiales	Burkholderiaceae	Ottowia	Unclassified Ottowia	CA	AS	Week 2
merD	Burkholderiales	Burkholderiaceae	Ottowia	Unclassified Ottowia	CA	AS	Week 2
merE	Burkholderiales	Burkholderiaceae	Ottowia	Unclassified Ottowia	CA	AS	Week 2
merP	Burkholderiales	Burkholderiaceae	Ottowia	Unclassified Ottowia	CA	AS	Week 2
merR	Burkholderiales	Burkholderiaceae	Ottowia	Unclassified Ottowia	CA	AS	Week 2
merT	Burkholderiales	Burkholderiaceae	Ottowia	Unclassified Ottowia	CA	AS	Week 2
sul1	Competibacterales	Competibacteraceae	Competibacter	Competibacter phosphatis	CA	AS	Week 2
tet(X2)	Chitinophagales	Chitinophagaceae	Edaphocola	Unclassified Edaphocola	CA	AS	Week 2
blaOXA	Burkholderiales	Rhodocyclaceae	Rugosibacter	Rugosibacter sp002422995	CA	AS	Week 3
mcr-5	Pseudomonadales	Moraxellaceae	UBA2031	Unclassified UBA2031	CA	AS	Week 3
merA	Burkholderiales	Burkholderiaceae	JADKHE01	Unclassified JADKHE01	CA	AS	Week 3
merC	Burkholderiales	Burkholderiaceae	JADKHE01	Unclassified JADKHE01	CA	AS	Week 3
merC	Burkholderiales	Burkholderiaceae	Ottowia	Unclassified Ottowia	CA	AS	Week 3

merD	Burkholderiales	Burkholderiaceae	Ottowia	Unclassified Ottowia	CA	AS	Week 3
merE	Burkholderiales	Burkholderiaceae	Ottowia	Unclassified Ottowia	CA	AS	Week 3
merP	Burkholderiales	Burkholderiaceae	Ottowia	Unclassified Ottowia	CA	AS	Week 3
merR	Burkholderiales	Burkholderiaceae	JADKHE01	Unclassified JADKHE01	CA	AS	Week 3
merR	Burkholderiales	Burkholderiaceae	Ottowia	Unclassified Ottowia	CA	AS	Week 3
merT	Burkholderiales	Burkholderiaceae	JADKHE01	Unclassified JADKHE01	CA	AS	Week 3
merT	Burkholderiales	Burkholderiaceae	Ottowia	Unclassified Ottowia	CA	AS	Week 3
sul1	Competibacterales	Competibacteraceae	Competibacter	Competibacter phosphatis	CA	AS	Week 3
mcr-5	Pseudomonadales	Moraxellaceae	UBA2031	Unclassified UBA2031	CA	Effluent	Week 3
kefB-GI	Burkholderiales	Burkholderiaceae	PHCI01	PHCI01 sp002842205	CA	Effluent	Week 3
psi-GI	Burkholderiales	Burkholderiaceae	PHCI01	PHCI01 sp002842205	CA	Effluent	Week 3
trxLHR	Burkholderiales	Burkholderiaceae	PHCI01	PHCI01 sp002842205	CA	Effluent	Week 3
mph(E)	Pseudomonadales	Moraxellaceae	UBA2031	Unclassified UBA2031	CA	Effluent	Week 3
msr(E)	Pseudomonadales	Moraxellaceae	UBA2031	Unclassified UBA2031	CA	Effluent	Week 3
sul1	Burkholderiales	Thiobacillaceae	Thiobacillus	Unclassified Thiobacillus	CA	Effluent	Week

3.2 List of all resistance genes detected and number of times detected

Gene ID	*n*
aac(3)-I	2
aac(3)-IId	2
aac(6')-IIa	2
aac(6')-Ib4	2
aadA1	4
aadA10	3
aadA11	4
aadA13	1
aadA2	7
aadA27	3
aadA5	5
aadA6	1
aadA7	4
aadE	3
aadS	10
abaF	4
acrF	4
ant(2'')-Ia	6
aph(3'')-Ib	22
aph(3')-IIIa	2
aph(3')-IIa	1
aph(3')-IIb	2
aph(3')-Ia	4
aph(3')-Ib	1
aph(3')-XV	2
aph(6)-Id	19
arr	3
arr-2	2
arsA	11
arsB	10
arsC	14
arsD	9
arsR	12
asr	2
bexA	3
bla	21

blaACT	5
blaACT-52	1
blaAER	9
blaBES	2
blaBRO	1
blaBRO-2	2
blaCARB-16	1
blaCMH	4
blaCMY	5
blaCMY-48	1
blaCTX-M	2
blaEC	5
blaFONA	1
blaFOX	3
blaGES-1	4
blaGES-5	9
blaGES-6	3
blaGES-7	3
blaIDC	2
blaIMP-18	1
blaKPC	1
blaKPC-2	9
blaLCR	1
blaLHK	6
blaM	2
blaMCA	6
blaMOX	8
blaMOX-9	3
blaNPS	2
blaNPS-1	1
blaOXA	81
blaOXA-1	2
blaOXA-10	4
blaOXA-119	1
blaOXA-129	1
blaOXA-2	4
blaOXA-347	6
blaOXA-4	2
blaOXA-5	2
blaOXA-732	1

blaOXA-9	6
blaOXA-912	1
blaOXY	3
blaPAC	4
blaPAU	8
blaPER	2
blaRCP	6
blaRSC1	1
blaRSD2	4
blaRTG	1
blaRm3	2
blaSHV	3
blaTEM	7
blaTEM-1	1
blaTLA	5
blaTLA-1	1
blaVEB	4
blaVEB-1	5
blaVEB-9	2
bleO	1
catA16	2
catB3	5
cblA	4
cepH	2
cepS	1
cfxA	9
cfxA6	1
chrA	11
cmlA1	2
cmlA5	12
cmx	1
copA	1
copB	3
copL	1
copR	12
copS	3
cphA	5
cphA1	3
dfrA1	4
dfrA12	1

dfrA14	3
dfrA3	2
dfrB5	2
dfrF	1
dpsA	1
emhA	3
emhB	1
emrD	9
emrE	2
ere(A)	15
ere(D)	6
erm(B)	3
erm(F)	7
erm(G)	4
espX1	1
fdeC	1
fieF	16
floR	4
floR2	4
fosA	5
hdeD-GI	13
hsp20	6
icr-Mo	5
iss	1
kdeA	1
kefB-GI	13
lnu(AN2)	6
lnu(B)	6
lnu(C)	7
lnu(F)	5
lpfA	1
lsa(E)	5
mcr-10.1	2
mcr-3	3
mcr-4	1
mcr-5	5
mcr-5.1	6
mcr-9	1
mdtM	3
mef(A)	10

mef(B)	9
mef(C)	17
mef(En2)	7
merA	35
merB	5
merC	24
merD	59
merE	60
merF	24
merP	97
merR	105
merT	107
mph(A)	22
mph(E)	23
mph(F)	8
mph(G)	13
mreA	6
msr(D)	5
msr(E)	31
ncrA	4
ncrB	4
ncrC	3
ncrY	3
norM	1
oqxA	6
oqxB	1
papA	1
pcoA	9
pcoB	11
pcoC	11
pcoD	8
pcoE	1
pcoR	8
pcoS	8
psi-GI	8
qac	2
qacEdelta1	3
qacG2	5
qacK	1
qacL	10

qnrB19	5
qnrS	1
qnrS2	9
qnrVC1	5
qnrVC4	10
qnrVC6	1
shsP	14
silA	3
silB	10
silC	9
silE	7
silF	9
silP	8
silR	9
silS	8
sul1	20
sul2	12
terB	1
terC	2
terD	6
terE	1
terW	3
terZ	4
tet(32)	2
tet(36)	5
tet(39)	9
tet(40)	2
tet(A)	15
tet(C)	24
tet(E)	10
tet(G)	9
tet(M)	3
tet(O)	7
tet(Q)	13
tet(S)	1
tet(W)	10
tet(X1)	4
tet(X2)	17
tla2	1
tmexC1	1

tmexD1	1
toprJ1	5
trxLHR	14
ybtP	1
ybtQ	1
yfdX1	17
yfdX2	18
ymgB	2
